# Gradient Methods on Strongly Convex Feasible Sets and Optimal Control of Affine Systems

**DOI:** 10.1007/s00245-018-9528-3

**Published:** 2018-10-06

**Authors:** V. M. Veliov, P. T. Vuong

**Affiliations:** Institute of Statistics and Mathematical Methods in Economics, Vienna University of Technology, Vienna, Austria

**Keywords:** Optimal control, Mathematical programming, Numerical methods, Gradient methods, Affine control systems, Bang–bang control, 49M25, 90C25, 90C48, 49M37

## Abstract

The paper presents new results about convergence of the gradient projection and the conditional gradient methods for abstract minimization problems on strongly convex sets. In particular, linear convergence is proved, although the objective functional does not need to be convex. Such problems arise, in particular, when a recently developed discretization technique is applied to optimal control problems which are affine with respect to the control. This discretization technique has the advantage to provide higher accuracy of discretization (compared with the known discretization schemes) and involves strongly convex constraints and possibly non-convex objective functional. The applicability of the abstract results is proved in the case of linear-quadratic affine optimal control problems. A numerical example is given, confirming the theoretical findings.

## Introduction

Solving numerically optimal control problems in which the control function appears linearly, and performing error analysis, are still challenging issues due to the typical discontinuity of the optimal control. Considerable progress was made in the past decade in the analysis of discretization schemes in combination with various methods of solving the resulting discrete-time optimization problems. The papers [1, 2, 25, 27] apply to problems with linear dynamics, while [3, 11] address nonlinear affine (in the control) dynamics. Usually the discretization is performed by Runge–Kutta schemes (mainly the Euler scheme) and the accuracy is at most of first order due to the discontinuity of the optimal control. Discretization schemes of higher accuracy were recently proposed in [21, 24] for systems with linear dynamics and Mayer or Bolza problems. In both cases the error analysis is based on the assumption that the optimal control is of purely bang–bang type.

On the other hand, the papers [12, 23] present convergence results for a version of the (abstract) Newton method for nonlinear problems, affine with respect to the control. Every step of the Newton method requires solving a linear-quadratic (affine in the control) optimal control problem for a linear system, namely a problem of the following type:

1 $$\begin{aligned} \mathop {{\mathrm{minimize}}}\limits _{x,u} \, J(x,u):= & {} \frac{1}{2} x(T)^\top Qx(T)+q^\top x(T)\nonumber \\&+\int _0^T \left( \frac{1}{2}x(t)^\top W(t)x(t)+x(t)^\top S(t)u(t) \right) \!{\mathrm{\,d}}t, \end{aligned}$$

subject to

2 $$\begin{aligned} \dot{x}(t)= & {} A(t)x(t)+B(t)u(t)+d(t), \quad x(0) = x_0, \quad t\in [0,T], \end{aligned}$$

3 $$\begin{aligned}&u(t)\in U:=[-1,1]^m. \end{aligned}$$

Here, [0, *T*] is a fixed time horizon, $Q\in \mathbb {R}^{n\times n}, q \in \mathbb {R}^n$, $A(t), W(t)\in \mathbb {R}^{n\times n}$, $B(t),S(t)\in \mathbb {R}^{n\times m}$, $d(t)\in \mathbb {R}^{n}$ for every $t\in [0,T]$, the superscript $\top$ means transposition. Admissible controls are all measurable functions $u:[0,T]\rightarrow U$. The state of the system at time *t* is $x(t)\in \mathbb {R}^n$, where $x(\cdot )$ is the (absolutely continuous) solution of (2), given an admissible control $u(\cdot )$. Linear terms are not included in the integrand in (1), since they can be shifted in a standard way into the differential equation (2).

For solving the above problem one can apply the high-order discretization scheme developed in [21, 24]. It results in a discrete-time optimal control problem (a mathematical programming problem), where the gradient of the objective function can be calculated following a standard procedure involving the solution of the associated adjoint system, so that gradient-type methods are conveniently applicable. And here we encounter a remarkable fact: although neither the objective functional (1) of the continuous-time problem (1)–(3) nor the control constraints (3) are strongly convex, it turns out that the feasible set of the discretized problem is strongly convex. This brings into consideration the issue of convergence of gradient methods for problems with strongly convex feasible sets and possibly non-convex objective functions (even if the functional *J* in (1) is convex on the set of admissible control–trajectory pairs, the discretized problem may fail to be convex!).

Versions of the gradient projection method (GPM) and the conditional gradient method (CGM) are widely studied (see e.g. [18, 19] and the references therein), but results about linear convergence of the generated sequence of iterates seem to be available only for problems with strongly convex objective functions. Exceptions are the papers [6, 15], where strong convexity is assumed for the feasible set instead of the objective function. However, as clarified in the end of Sect. 2.1 below, the additional assumptions in these two papers are rather strong and are not fulfilled for the problem arising in the optimal control context as described above.

In this paper we present convergence results for the gradient projection and the conditional gradient methods for minimization problems in a Hilbert space, where the feasible set is strongly convex but the objective functional is not necessarily convex. These results are new even for convex or strongly convex objective functional, but we relax the convexity assumption due to the needs of our main goal—to cover the problems arising in optimal control of affine systems, as described above. For that we consider objective functionals that we called, for shortness, $(\varepsilon ,\delta )$-approximately convex. These functions constitute a larger class than that of the weakly convex functions (see e.g. [4]). In Sect. 2.1 we prove linear convergence of the sequence of approximate solutions generated by the GPM, provided that the step sizes are appropriately chosen. Apart from the applicability for non-convex objective functionals, this result does not require the additional conditions in [6, 15]. As usual, the “appropriate” choice of the step sizes is expressed by some constants related to the data of the problem, which are often not available (or very roughly estimated). Therefore, we present an additional convergence result involving a rather general and constructive condition for the step sizes (well-known in the literature).

The conditional gradient method may have some advantages (compared with the GPM) in our optimal control application. For this reason we also prove a linear convergence result for the CGM. This is done in Sect. 2.2.

In Sect. 3 we turn back to the optimal control problem (1)–(3). The first two subsections are preliminary, where we introduce notations, formulate assumptions and present the discrete approximation introduced in [21, 24] and the error estimate proved in [24]. All this is needed for understanding of the implementation of the GPM and the CGM and of the proofs of the error estimations. Then, in Sects. 3.3 and 3.4 we prove the applicability of the abstract convergence results, obtained in Sect. 2, to our discretized optimal control problem and present details about the implementation of the GPM and the CGM. A numerical example that confirms the theoretical findings is given in Sect. 3.5.

The paper concludes with indication of some open problems for further research (Sect. 4).

## Gradient Methods for Problems with Strongly Convex Feasible Set

In this section we investigate the convergence of certain gradient methods for an abstract minimization problem of the form

4 $$\begin{aligned} \min _{w \in K} f(w), \end{aligned}$$

where *K* is a convex subset of a real Hilbert space *H* and $f : H \rightarrow {\mathbb {R}}$ is a function for which certain conditions weaker than convexity will be posed. We remind that if $w^* \in K$ is a (local) solution of (4) and *f* is Fréchet-differentiable at $w^*$ then

$$\begin{aligned} \left\langle \nabla f(w^*), y-w^* \right\rangle \ge 0 \quad \forall y \in K. \end{aligned}$$

Convergence results for gradient projection methods for this problem in finite dimensional spaces and convex *f* are known (see e.g. [19]). It has been proved that the iterative sequence generated by versions of the gradient projection method converges linearly to a solution, provided that the objective function *f* is strongly convex and its gradient is Lipschitz continuous. Extensions to infinite dimensional Hilbert spaces are straightforward. In contrast, in our results below the function *f* does not even need to be convex, while the set *K* is assumed strongly convex. Some convergence results for smooth convex functions *f* and strongly convex sets *K* are obtained in [6, 15], but under suppositions that (apart from the convexity of *f*) are not satisfied in our main motivation as described in the introduction (see Remark 2.3 below). The convergence results presented in this section are substantially stronger.

As usual, $\left<\cdot ,\cdot \right>$ denotes the inner product in *H* and $\Vert \cdot \Vert$—the induced norm.

Let *K* be a nonempty closed convex subset of *H*. For each $u\in H$, there exists a unique point in *K* (see [16, p. 8]), denoted by $P_K(u)$, such that

$$\begin{aligned} \Vert u-P_K(u)\Vert \le \Vert u-v\Vert \quad \forall v\in K. \end{aligned}$$

It is well-known that the metric projection $P_K$ is a nonexpansive mapping, i.e., for all $u,v\in H$

$$\begin{aligned} \Vert P_K(u)-P_K(v)\Vert \le \Vert u-v\Vert . \end{aligned}$$

Moreover for any $u \in H$ and $v\in K$, it holds that

5 $$\begin{aligned} \left<u-P_K(u), v-P_K(u)\right>\le 0. \end{aligned}$$

Conversely, if $w\in K$ and $\left<u-w, v-w\right>\le 0$ for all $v\in K$, then $w=P_K(u)$.

Below we remind the following notions.

### Definition 2.1

The set $K \subset H$ is called *strongly convex* or $\gamma$-strongly convex if there exists a number $\gamma > 0$ (called modulus of strong convexity) such that for any $u,v \in K$ and any $\lambda \in [0,1]$ it holds that

$$\begin{aligned} \lambda u+(1-\lambda ) v + \lambda (1-\lambda )\frac{\gamma }{2} \Vert u-v\Vert ^2 z \in K \quad \forall \, z \text{ with } \Vert z\Vert \le 1. \end{aligned}$$

An alternative definition is often used in the literature: a set is strongly convex (with respect to the number $R > 0$) if it coincides with the intersection of all balls of radius *R* containing this set. The two definitions are equivalent (see e.g. [28, Theorem 1]) and the relation between $\gamma$ and *R* is that $R = 1/\gamma$.^1^

### Definition 2.2

A function $f: H \rightarrow {\mathbb {R}}$ is called *L-smooth* on *K* if *f* is Fréchet differentiable and its derivative, $\nabla f$, is *L*-Lipschitz continuous on *K*, i.e.,

$$\begin{aligned} \Vert \nabla f(u)-\nabla f(v)\Vert \le L \Vert u-v\Vert \qquad \forall \, u,v \in K. \end{aligned}$$

The following definition introduces a property that is usually called “weak convexity” or “paraconvexity” (see e.g. [4]).

### Definition 2.3

A function $f: H \rightarrow {\mathbb {R}}$ is called $\varepsilon$*-convex* (with $\varepsilon \ge 0$) on a convex subset $K \subset H$ at $\hat{w} \in K$ if the function $f_\varepsilon (w) := f(w) + \frac{1}{2} \varepsilon \Vert w - \hat{w} \Vert ^2$ is convex on *K* at $\hat{w}$, i.e.

$$\begin{aligned} f_\varepsilon (\alpha w + (1 - \alpha )\hat{w} ) \le \alpha f_\varepsilon (w) + (1-\alpha ) f_\varepsilon (\hat{w} ) \end{aligned}$$

for every $w \in K$ and $\alpha \in (0,1)$.

If $f:H \rightarrow {\mathbb {R}}$ is $\varepsilon$-convex at $\hat{w}$ and differentiable, then

$$\begin{aligned} \langle \nabla f_\varepsilon (w) - \nabla f_\varepsilon (\hat{w} ), w - \hat{w} \rangle \ge 0 \qquad \forall \, w \in K. \end{aligned}$$

This implies that

$$\begin{aligned} \langle \nabla f(w) - \nabla f(\hat{w} ), w - \hat{w} \rangle \ge - \varepsilon \Vert w - \hat{w} \Vert ^2 \qquad \forall \, w \in K. \end{aligned}$$

In our main application, the function *f* does not need to be even $\varepsilon$-convex with $\varepsilon$ reasonably small. Therefore we further weaken the convexity as in the following definition.

### Definition 2.4

A Fréchet-differentiable function $f: H \rightarrow {\mathbb {R}}$ is called $(\varepsilon ,\delta )$*-approximately convex* (with $\varepsilon , \delta \ge 0$) on a convex subset $K \subset H$ at $\hat{w} \in K$ if

6 $$\begin{aligned} \langle \nabla f(w) - \nabla f(\hat{w} ), w - \hat{w} \rangle \ge - \varepsilon \Vert w - \hat{w} \Vert ^2 \qquad \forall \, w \in K \quad \text{ with }\quad \Vert w-\hat{w} \Vert \ge \delta . \end{aligned}$$

Notice that $\delta$ can be taken equal to zero in the above definition, in which case the $(\varepsilon ,\delta )$-approximate convexity reduces to $\varepsilon$-convexity.

The following three results provide the ground for the error analysis of the GPM and the CGM.

### Proposition 2.1

Assume that *K* is $\gamma$-strongly convex, *f* is differentiable on *K* and $\hat{w} \in K$ is a solution of problem (4) such that $\,\Vert \nabla f(\hat{w} )\Vert \ge \rho$ for some number $\rho > 0$. Assume also that *f* is $(\varepsilon ,\delta )$-approximately convex on *K* at $\hat{w}$ and that the number $\nu := \frac{\gamma \rho }{4} - \varepsilon$ is positive. Then

7 $$\begin{aligned} \left\langle \nabla f(w), w-\hat{w} \right\rangle \ge \nu \Vert w-\hat{w} \Vert ^2 \qquad \forall \, w \in K \text{ with } \Vert w-\hat{w} \Vert \ge \delta . \end{aligned}$$

Moreover, any solution of problem (4) is at distance at most $\delta$ from $\hat{w}$.

### Proof

Setting $z=\frac{-\nabla f(\hat{w} )}{\Vert \nabla f(\hat{w} )\Vert }$, we have $\Vert z\Vert =1$. By the strong convexity of *K* we obtain that for any $w \in K$

$$\begin{aligned} y := \frac{1}{2}(w+\hat{w} )+\frac{\gamma }{8} \Vert w-\hat{w} \Vert ^2 z \in K. \end{aligned}$$

Due to (6), for all $w \in K$ with $\Vert w-\hat{w} \Vert \ge \delta$ we have

$$\begin{aligned} \left\langle \nabla f(w)- \nabla f(\hat{w} ), w-\hat{w} \right\rangle \ge - \varepsilon \Vert w - \hat{w} \Vert ^2. \end{aligned}$$

Hence,

8 $$\begin{aligned} \left\langle \nabla f(w) , w-\hat{w} \right\rangle\ge & {} \left\langle \nabla f(\hat{w} ), w-\hat{w} \right\rangle - \varepsilon \Vert w - \hat{w} \Vert ^2\nonumber \\= & {} 2\left\langle \nabla f(\hat{w} ), \frac{w+\hat{w} }{2} -y \right\rangle +2\left\langle \nabla f(\hat{w} ), y-\hat{w} \right\rangle - \varepsilon \Vert w - \hat{w} \Vert ^2.\nonumber \\ \end{aligned}$$

The optimality of $\hat{w}$ implies that

$$\begin{aligned} \left\langle \nabla f(\hat{w} ), y-\hat{w} \right\rangle \ge 0. \end{aligned}$$

Then from (8) we obtain that

$$\begin{aligned} \left\langle \nabla f(w) , w-\hat{w} \right\rangle\ge & {} 2\left\langle \nabla f(\hat{w} ), \frac{\gamma }{8} \Vert w-\hat{w} \Vert ^2 \frac{ \nabla f(\hat{w} )}{\Vert \nabla f(\hat{w} )\Vert }\right\rangle - \varepsilon \Vert w - \hat{w} \Vert ^2\nonumber \\= & {} \frac{\gamma }{4} \Vert \nabla f(\hat{w} )\Vert \Vert w-\hat{w} \Vert ^2 - \varepsilon \Vert w - \hat{w} \Vert ^2 \ge \nu \Vert w-\hat{w} \Vert ^2, \end{aligned}$$

that is, (7).

Now assume that $\bar{w}$ is another solution of (4). The optimality of $\bar{w}$ implies, in particular, that

$$\begin{aligned} \left\langle \nabla f(\bar{w}), \hat{w} -\bar{w} \right\rangle \ge 0. \end{aligned}$$

Assuming that $\Vert \bar{w} - \hat{w} \Vert > \delta$ we may substitute $w=\bar{w} \in K$ in (7), which gives

$$\begin{aligned} \left\langle \nabla f(\bar{w}), \bar{w}-\hat{w} \right\rangle \ge \nu \Vert \bar{w}-\hat{w} \Vert ^2. \end{aligned}$$

Adding the last two inequalities we obtain that

$$\begin{aligned} 0 \ge \nu \Vert \bar{w}-\hat{w} \Vert ^2. \end{aligned}$$

which contradicts the assumption $\Vert \bar{w} - \hat{w} \Vert > \delta$. The proof is completed. $\square$

Property (7) will play an important role in the further analysis. In fact, the $(\varepsilon ,\delta )$-approximate convexity of *f* and the strong convexity of *K* were needed just to ensure existence of $\nu > 0$ and $\delta \ge 0$ for which condition (7) is fulfilled. We mention that (7) is always fulfilled if the set *K* is convex and the function*f* is strongly convex, which is not the case here.

### Lemma 2.1

Let *f* be differentiable on *K* and let condition (7) be fulfilled with some $\nu > 0$. If for some $w \in K$ and $\lambda > 0$ it holds that $P_K(w - \lambda \nabla f(w)) = w$, then $\Vert w - \hat{w}\Vert \le \delta$.

### Proof

Contrary to the claim of the lemma, assume that $\Vert w - \hat{w}\Vert > \delta$. Then from Proposition 2.1 we have that the first inequality in (7) is fulfilled by *w*. From the condition $P_K(w - \lambda \nabla f(w)) = w$ we have that

$$\begin{aligned} \langle \nabla f(w), u - w \rangle \ge 0 \qquad \forall u \in K. \end{aligned}$$

Applying this inequality for $u = \hat{w}$ and adding it to the first inequality in (7) we obtain that

$$\begin{aligned} 0 \ge \nu \Vert w - \hat{w}\Vert ^2, \end{aligned}$$

which is a contradiction. $\square$

### Lemma 2.2

Let *f* be differentiable on *K* and let condition (7) be fulfilled with some $\nu > 0$. If for some $w \in K$ it holds that $\nabla f(w) = 0$, then $\Vert w - \hat{w}\Vert \le \delta$.

### Proof

If we assume $\Vert w - \hat{w}\Vert > \delta$, then from the first inequality in (7) we have

$$\begin{aligned} 0 \ge \nu \Vert w - \hat{w} \Vert ^2, \end{aligned}$$

which is a contradiction. $\square$

### The Gradient Projection Method

For solving the minimization problem (4), we consider first the most classical algorithm, the gradient projection method (GPM) stated below. In the formulation of the algorithm we only assume that *f* is *L*-smooth.

**Algorithm GPM**.

- **Step 0:** Choose $w_0\in K$. Set $k=0$.
- **Step 1:** If $w_{k}=P_K\left( w_k- \nabla f(w_k)\right)$ then Stop. Otherwise, go to Step 2.
- **Step 2:** Choose $\lambda _k >0$ and calculate
  9 $$\begin{aligned} w_{k+1}=P_K \left( w_k-\lambda _k \nabla f(w_k)\right) . \end{aligned}$$
  Replace *k* by $k+1$; go to Step 1.

It is well-known that for convex *f* and *K* the GPM has the error estimate $O(\frac{1}{k})$ in term of the objective function when $\lambda _k =\lambda \in (0, \frac{1}{L}]$, see e.g. [7]. More precisely, if problem (4) has a solution and $\hat{f}$ is the minimal value of *f* on *K*, then

$$\begin{aligned} f(w_k) - \hat{f} \le \frac{L m_0}{2k} \qquad \forall k, \end{aligned}$$

where $m_0$ is the distance from $w_0$ to the solution set of (4). If in addition, *f* is strongly convex, then the sequence $\left\{ w_k \right\}$ converges linearly to the unique solution of (4). If *f* is only convex (but not necessarily strongly convex), the sequence $\left\{ w_k \right\}$ converges weakly [20]. When *K* is strongly convex, the linear convergence of $\left\{ w_k \right\}$ is obtained under additional conditions (too strong for our main application) in [5, 6, 15].

In this subsection, we prove that if condition (7) is fulfilled with $\nu > 0$ then the sequence $\left\{ w_k \right\}$ generated by the GPM linearly approaches $\hat{w}$ at least until entering a $\delta$-neighborhood of $\hat{w}$. Proposition 2.1 gives conditions for existing of such $\nu$ in terms of strong convexity of the set *K* and $(\varepsilon ,\delta )$-approximate convexity of the function *f*. We mention that if the above algorithm of the GPM stops at Step 1 for some *k* then, according to Lemma 2.1, $\Vert w_k - \hat{w}\Vert \le \delta$, that is, a $\delta$-approximate solution is attained (obviously this is meaningful only if $\delta$ is sufficiently small).

#### Proposition 2.2

Let *f* be *L*-smooth on *K*, let condition (7) be fulfilled with some $\nu > 0$, and let $\Vert w_0 - \hat{w} \Vert \ge \delta$. Then the sequence $\left\{ w_k \right\}$ generated by the GPM satisfies the inequality

10 $$\begin{aligned} \left[ 1+\lambda _k \left( 2\nu -\lambda _k L^2 \right) \right] \Vert w_{k+1}-\hat{w} \Vert ^2\le \Vert w_k-\hat{w} \Vert ^2 \end{aligned}$$

at least as long as $\Vert w_{k+1}-\hat{w} \Vert \ge \delta$.

#### Proof

Since $w_{k+1}=P_K(w_k-\lambda _k \nabla f(w_k))$, due to inequality (5) we have

$$\begin{aligned} \langle w_k-\lambda _k \nabla f(w_k)-w_{k+1}, w - w_{k+1}\rangle \le 0 \quad \forall w\in K. \end{aligned}$$

Substitution of $w=\hat{w} \in K$ in this inequality yields

$$\begin{aligned} \langle w_k-\lambda _k \nabla f(w_k)-w_{k+1}, \hat{w} - w_{k+1}\rangle \le 0, \end{aligned}$$

or equivalently

11 $$\begin{aligned} 2\langle w_k-w_{k+1}, \hat{w} - w_{k+1}\rangle\le & {} 2\lambda _k\langle \nabla f(w_k), \hat{w} -w_{k+1}\rangle \nonumber \\= & {} -2\lambda _k\langle \nabla f(w_{k+1}), w_{k+1}-\hat{w} \rangle \nonumber \\&+\, 2\lambda _k\langle \nabla f(w_k)-\nabla f(w_{k+1}), \hat{w} -w_{k+1}\rangle . \end{aligned}$$

Since $w_{k+1} \in K$ and $\lambda _k >0$, if $\Vert w_{k+1}-\hat{w} \Vert \ge \delta$ then due to (7)

12 $$\begin{aligned} -2\lambda _k \langle \nabla f(w_{k+1}), w_{k+1}-\hat{w} \rangle \le -2\lambda _k \nu \Vert w_{k+1}-\hat{w} \Vert ^2. \end{aligned}$$

By the Cauchy–Schwarz inequality and the Lipschitz continuity of $\nabla f$, we obtain that

13 $$\begin{aligned} 2\lambda _k\langle \nabla f(w_k)-\nabla f(w_{k+1}), \hat{w} -w_{k+1}\rangle\le & {} 2\lambda _k\Vert \nabla f(w_k)-\nabla f(w_{k+1})\Vert \Vert w_{k+1}-\hat{w} \Vert \nonumber \\\le & {} 2\lambda _k L\Vert w_k-w_{k+1}\Vert \Vert w_{k+1}-\hat{w} \Vert \nonumber \\\le & {} \Vert w_k-w_{k+1}\Vert ^2+(\lambda _kL)^2\Vert w_{k+1}-\hat{w} \Vert ^2.\nonumber \\ \end{aligned}$$

Inequalities (11), (12) and (13) imply that

14 $$\begin{aligned} 2\langle w_k-w_{k+1}, \hat{w} - w_{k+1}\rangle \le - 2 \lambda _k \nu \Vert w_{k+1}-\hat{w} \Vert ^2+ \Vert w_k- w_{k+1}\Vert ^2+(\lambda _k L)^2\Vert w_{k+1}-\hat{w} \Vert ^2. \end{aligned}$$

On the other hand,

15 $$\begin{aligned} \begin{array}{lll} 2\langle w_k-w_{k+1}, \hat{w} - w_{k+1}\rangle= & {} \Vert w_{k}-w_{k+1}\Vert ^2+\Vert w_{k+1}-\hat{w} \Vert ^2-\Vert w_k-\hat{w} \Vert ^2. \end{array} \end{aligned}$$

Combining (14) and (15) we obtain that

$$\begin{aligned}&\Vert w_{k}-w_{k+1}\Vert ^2+\Vert w_{k+1}-\hat{w} \Vert ^2-\Vert w_k-\hat{w} \Vert ^2\\&\quad \le \, -2 \lambda _k \nu \Vert w_{k+1}-\hat{w} \Vert ^2 + \Vert w_k-w_{k+1}\Vert ^2+ (\lambda _k L)^2\Vert w_{k+1}-\hat{w} \Vert ^2, \end{aligned}$$

hence (10) is satisfied. $\square$

Now we can state and prove the main convergence result for the GPM.

#### Theorem 2.1

Let all the assumptions in Proposition 2.2 be satisfied. Let the sequence $\left\{ \lambda _k\right\}$ be chosen such that

16 $$\begin{aligned} 0<a\le \lambda _k\le b< \frac{2\nu }{L^2} \quad \forall k, \end{aligned}$$

where *a*, *b* are some positive constants. Define

17 $$\begin{aligned} \mu =\frac{1}{\sqrt{1+a \left( 2 \nu - bL^2 \right) }}\in (0,1). \end{aligned}$$

Let $\{w_k\}$ be the sequence generated by the GPM. Then for every *k*, if $\Vert w_{k+1}-\hat{w} \Vert \ge \delta$ then

18 $$\begin{aligned} \Vert w_{k+1}-\hat{w} \Vert \le \mu \,\Vert w_k-\hat{w} \Vert . \end{aligned}$$

Moreover, for every *k*, if $\Vert w_{i+1}-\hat{w} \Vert \ge \delta$, $i=0, \ldots , k$, then the following a priori and a posteriori error estimates hold:

19 $$\begin{aligned} \Vert w_{k+1}-\hat{w} \Vert \le \frac{\mu ^{k+1}}{1-\mu }\Vert w_1-w_0\Vert , \end{aligned}$$

and

20 $$\begin{aligned} \Vert w_{k+1}-\hat{w} \Vert \le \frac{\mu }{1-\mu }\Vert w_{k+1}-w_k\Vert . \end{aligned}$$

Before proving the theorem we mention that in the case of an $\varepsilon$-convex function *f* (that is, if $\delta = 0$) the first claim of the theorem means that the sequence generated by the GPM converges linearly to the (unique) solution $\hat{w}$. In the case $\delta > 0$ we also have linear convergence at least until the generated sequence enters the $\delta$-neighborhood of $\hat{w}$. Thus in this case the theorem is meaningful only if $\delta$ is reasonably small.

#### Proof

It follows from (16) that $[1+\lambda _k(2 \nu -\lambda _kL^2)]\ge [1+a(2 \nu - bL^2)]>1$ for all *k*. By (10) and the above inequalities,

$$\begin{aligned} \left[ 1+a\left( 2\nu - bL^2 \right) \right] \Vert w_{k+1}-\hat{w} \Vert ^2\le \Vert w_k-\hat{w} \Vert ^2, \end{aligned}$$

provided that $\Vert w_{k+1}-\hat{w} \Vert \ge \delta$. Hence

21 $$\begin{aligned} \Vert w_{k+1}-\hat{w} \Vert \le \mu \Vert w_k-\hat{w} \Vert \end{aligned}$$

with $\mu \in (0,1)$ being defined by (17).

The proof of (19) and (20) is standard, but we present it for completeness. By (21),

$$\begin{aligned} \Vert w_{k+1}-\hat{w} \Vert \le \mu \Vert w_k-\hat{w} \Vert \le \mu ^2\Vert w_{k-1}-\hat{w} \Vert \le \cdots \le \mu ^{k+1}\Vert w_0-\hat{w} \Vert . \end{aligned}$$

Observe that

$$\begin{aligned} \Vert w_k-\hat{w} \Vert \le \Vert w_k-w_{k+1}\Vert +\Vert w_{k+1}-\hat{w} \Vert \le \Vert w_k-w_{k+1}\Vert +\mu \Vert w_k-\hat{w} \Vert , \end{aligned}$$

and so $\Vert w_k-\hat{w} \Vert \le \frac{1}{1-\mu }\Vert w_k-w_{k+1}\Vert$ for all *k*. Hence

$$\begin{aligned} w_{k+1}-\hat{w} \Vert\le & {} \mu ^{k+1}\Vert w_0-\hat{w} \Vert \le \frac{\mu ^{k+1}}{1-\mu }\Vert w_0-w_1\Vert ,\\ \Vert w_{k+1}-\hat{w} \Vert\le & {} \mu \Vert w_k-\hat{w} \Vert \le \frac{\mu }{1-\mu }\Vert w_k-w_{k+1}\Vert . \end{aligned}$$

$$\square $$

#### Remark 2.1

If the constants *L* and $\nu$ can be reasonably estimated, then inequalities (19) and (20) can be used to estimate the number of iterations of the GPM needed to achieve a given accuracy.

#### Remark 2.2

The value $\mu$ in (17) can be regarded as a function $\mu =\mu (a, b)$ of the variable (*a*, *b*) belonging to the domain

$$\begin{aligned} \left\{ (a, b)\in \mathbb {R}^2: 0<a\le b<\frac{2 \nu }{L^2}\right\} . \end{aligned}$$

It is a routine task to obtain that the minimum of $\mu (a,b)$ under the above constraints is achieved at $(a_*, b_*):=(\frac{\nu }{L^2}, \frac{\nu }{L^2})$ and the minimal value is $\mu _*:=\frac{L}{\sqrt{L^2+\nu ^2}}$. Hence, $\lambda _k=\frac{\nu }{L^2}$ would be an optimal choice of $\lambda _k$.

Since the parameters *L* and $\nu$ are usually not known in advance, we can consider the step size sequence $\left\{ \lambda _k \right\}$ as any non-summable converging to zero sequence of positive real numbers as it follows in the next theorem.

#### Theorem 2.2

Let the assumptions in Proposition 2.2 be satisfied. Let $\{\lambda _k\}$ be a sequence of positive scalars such that

22 $$\begin{aligned} \sum _{k=0}^{\infty }\lambda _k=+\infty , \quad \lim _{k\rightarrow \infty }\lambda _k=0. \end{aligned}$$

Then for every positive number $\delta ' \ge \delta$ all elements of the sequence $\{w_k\}$ with sufficiently large *k* are contained in the $\delta '$-neighborhood of $\hat{w}$. Moreover, there exists a natural number $k_0$ such that for each $k\ge k_0$ for which $\Vert w_{i+1}-\hat{w} \Vert \ge \delta$ is fulfilled for $i = k_0, \ldots , k$, it holds that $\lambda _k(2 \nu - \lambda _k L^2)>0$, and

23 $$\begin{aligned} \Vert w_{k+1}-\hat{w} \Vert \le \frac{1}{\sqrt{\prod _{i=k_0}^{k}[1+ \lambda _i(2\nu - \lambda _i L^2)]}}\Vert w_{k_0}-\hat{w} \Vert . \end{aligned}$$

Clearly, in the case $\delta = 0$ the first claim of the theorem implies strong convergence of the sequence $\{w_k\}$.

#### Proof

Since $\lambda _k\rightarrow 0$, there exists $k_0$ such that $4\lambda _kL^2<\gamma \rho -4 \varepsilon$ for every $k\ge k_0$. Hence,

$$\begin{aligned} \lambda _k\left( 2 \nu - \lambda _k L^2 \right)> \lambda _k \left( 2 \nu -\nu \right) =\nu \lambda _k >0, \end{aligned}$$

for all $k\ge k_0$. If *k* is such that $\Vert w_{i+1}-\hat{w} \Vert \ge \delta$, $i = k_0, \ldots , k$, then from (10) it follows that

$$\begin{aligned} \Vert w_{k+1}-\hat{w} \Vert ^2\le & {} \frac{1}{1+\lambda _k \left( 2 \nu -\lambda _kL^2 \right) }\Vert w_{k}-\hat{w} \Vert ^2\\\le & {} \frac{1}{\left[ 1+\lambda _k\left( 2 \nu -\lambda _kL^2 \right) \right] } \frac{1}{[1+\lambda _{k-1}(2 \nu - \lambda _{k-1}L^2)]}\Vert w_{k-1}-\hat{w} \Vert ^2\\&\vdots&\\\le & {} \frac{1}{\prod _{i=k_0}^{k}[1+\lambda _i(2 \nu -\lambda _iL^2)]}\Vert w_{k_0}-\hat{w} \Vert ^2, \end{aligned}$$

which proves (23).

Let us now prove the first claim of the theorem. For each *k* set

$$\begin{aligned} \alpha _k=\lambda _k\left( 2 \nu -\lambda _k L^2\right) \end{aligned}$$

and rewrite (23) (if it holds for *k*) as

24 $$\begin{aligned} \Vert w_{k+1}-\hat{w} \Vert \le \frac{1}{\sqrt{\prod _{i=k_0}^{k}(1+\alpha _i})}\Vert w_{k_0}-\hat{w} \Vert . \end{aligned}$$

Since $\alpha _k=\lambda _k(2 \nu -\lambda _kL^2)> \nu \lambda _k$ for each $k\ge k_0$, it follows from (22) that $\sum \nolimits _{k=k_0}^{\infty }\alpha _k=+\infty$. Hence

$$\begin{aligned} \displaystyle \prod _{i=k_0}^{k}(1+\alpha _i) \ge \displaystyle 1+\sum _{i=k_0}^{k}\alpha _i \longrightarrow +\infty \end{aligned}$$

as $k\rightarrow \infty$. Since (24) holds as long as $\Vert w_{i+1} - \hat{w} \Vert \ge \delta$ for $i=k_0,\ldots ,k$, we obtain that either $\Vert w_k - \hat{w} \Vert \longrightarrow 0$ or $\Vert w_k - \hat{w} \Vert < \delta$ for some $k \ge k_0$. If $\Vert w_k - \hat{w} \Vert \longrightarrow 0$ then the claim is true since $\delta ' > 0$. If $\Vert w_k - \hat{w} \Vert < \delta$ for some $k \ge k_0$, then $\Vert w_{k+1} - \hat{w} \Vert < \delta$. Indeed, if $\Vert w_{k+1} - \hat{w} \Vert \ge \delta$ then we have from (10)

$$\begin{aligned} \Vert w_{k+1}-\hat{w} \Vert ^2 \le \frac{1}{1+\alpha _k} \Vert w_{k}-\hat{w} \Vert ^2 < \delta ^2, \end{aligned}$$

which is a contradiction. Thus $w_k$ remains in the $\delta '$-neighborhood of $\hat{w}$ for all $k \ge k_0$. The proof is completed. $\square$

#### Remark 2.3

Using the contractivity of the projection onto strongly convex sets, Balashov and Golubev [6] and Golubev [15] obtained the linear convergence of the GPM for smooth, convex optimization problem with the following additional conditions:

(i) For any *k*, there exists a unit vector $n(w_k) \in N_K(w_k)$ such that
  $$\begin{aligned} \left\langle n(w_k), \nabla f(w_k) \right\rangle \le 0, \end{aligned}$$
  where $N_K(w_k)$ is the normal cone to *K* at $w_k$ defined as
  $$\begin{aligned} N_K(w_k):={\left\{ \begin{array}{ll} \emptyset &{} \text{ if } \quad w_k \notin K, \\ \{l\in H:\langle l,v-w_k \rangle \le 0\ \forall v\in K \} &{} \text{ if } \quad w_k\in K. \end{array}\right. } \end{aligned}$$
(ii) The problem (4) has a unique solution and it belongs to the boundary of *K*.

In our convergence analysis in Theorem 2.1, the assumptions (i), (ii) are eliminated, which is important for our main motivation (see the next section). Also important is that our result applies under the $(\varepsilon ,\delta )$-approximate convexity instead of convexity.

### The Conditional Gradient Method

In this subsection, we consider the conditional gradient method (CGM) for solving problem (4) with a $\gamma$-strongly convex set *K* and an $(\varepsilon ,\delta )$-approximate convex and *L*-smooth function *f*. This method dates back to the original work of Frank and Wolfe [13] which presented an algorithm for minimizing a quadratic function over a polytope using only linear optimization steps over the feasible set. The CGM for solving (strongly) convex problem was investigated in [8, 9, 14].

**Algorithm CGM**.

- **Step 0:** Choose $w_0\in K$. Set $k=0$.
- **Step 1:** If $\nabla f(w_k) = 0$, then Stop. Otherwise, find a solution $x_k$ of the problem
  25 $$\begin{aligned} \min _{y \in K} \, \left\langle \nabla f(w_k), y\right\rangle . \end{aligned}$$
- **Step 2:** If $x_k=w_k$, then Stop. Otherwise, go to Step 3.
- **Step 3:** If $\nabla f(w_k) \not = 0$, choose $\eta _k \in (0, \min \lbrace 1,\frac{\gamma \Vert \nabla f(w_k)\Vert }{4L}\rbrace ]$, calculate
  26 $$\begin{aligned} w_{k+1}=(1-\eta _k)w_k+\eta _k x_k, \end{aligned}$$
  replace *k* by $k+1$, and go to Step 1. Else the iteration process terminates.

Notice that if the above algorithm stops at Step 1 or Step 3 for some *k* then, under the assumptions of Lemma 2.2$\Vert w_k - \hat{w}\Vert \le \delta$, that is, an approximate solution is attained.

In general, problem (25) may fail to have a solution, in which case the CGM is not executable.

#### Remark 2.4

The objective function in the subproblem (25) in the CGM is linear, thus if *K* is a polytope, we encounter a linear programming problem which should be easier to solve than the quadratic programming subproblem (9) in the GPM. In the case considered in this paper the set *K* is not a polytope, thus (25) is not a linear programming problem. However, in our main application (see the next section) the set *K* is a product of (possibly large number of) simple two-dimensional strongly convex sets, so that (25) decomposes into two-dimensional subproblems that are easy to solve.

We will use the following global version of $(\varepsilon ,\delta )$-approximate convexity.

#### Definition 2.5

A Fréchet-differentiable function $f: H \rightarrow {\mathbb {R}}$ is called $(\varepsilon ,\delta )$*-approximately convex* on a convex subset $K \subset H$ if

27 $$\begin{aligned} f(w)-f(v) \ge \langle \nabla f(v), w - v \rangle - \frac{\varepsilon }{2} \Vert w - v \Vert ^2 \qquad \forall \, w,v \in K \text{ with } \Vert w-v \Vert \ge \delta . \end{aligned}$$

Clearly, (27) implies (6).

We begin the convergence analysis of the CGM with an inequality which will play a key role for obtaining convergence results. For convenience we assume that if the CGM terminates at some finite iteration $k =i$, (due to $\nabla f(w_i) = 0$) then the sequence $\{ w_k \}$ is extended as $w_k = w_i$ for $k > i$.

#### Proposition 2.3

Assume that *K* is $\gamma$-strongly convex, *f* is *L*-smooth on *K* and $\hat{w}$ is a solution of problem (4) such that $\Vert \nabla f(\hat{w} )\Vert \ge \rho$ for some number $\rho > 0$. Assume also that *f* is $(\varepsilon ,\delta )$-approximately convex on *K* and that the number $\nu :=\frac{\gamma \rho }{4}-\varepsilon$ is positive. Further, assume that at any iteration *k* a solution of the subproblem (25) does exist, and let $\{ w_k \}$ be the sequence generated by the CGM. Denote $\hat{f} := f(\hat{w} )$ and $\Delta _k := f(w_k) - \hat{f}$. Then

28 $$\begin{aligned} \Delta _{k+1} \le \left( 1-\frac{\nu \eta _k}{2 \nu +\varepsilon } \right) \Delta _k - \frac{\eta _k}{2}\left( \frac{\gamma \Vert \nabla f(w_k)\Vert }{4} - L \eta _k \right) \Vert x_{k}-w_k\Vert ^2, \end{aligned}$$

at least as long as $\Vert w_{k}-\hat{w} \Vert \ge \delta$.

#### Proof

If $\nabla f(w_i) = 0$ for some *i*, we have $x_k=w_k$ and $\Delta _k = 0$ for all $k \ge i$, hence (28). Thus we may assume that $\nabla f(w_k) \not = 0$ for the arbitrarily fixed *k* in the consideration below.

Since *f* is *L*-smooth on *K* we have (see, for example, [20, Lemma 1.30])

29 $$\begin{aligned} f(w_{k+1})\le & {} f(w_k)+\left\langle \nabla f(w_k), w_{k+1}-w_k \right\rangle +\frac{L}{2}\Vert w_{k+1}-w_k\Vert ^2 \nonumber \\= & {} f(w_k)+\eta _k \left\langle \nabla f(w_k), x_{k}-w_k \right\rangle +\frac{L}{2} \eta _k^2 \Vert x_{k}-w_k\Vert ^2. \end{aligned}$$

Subtracting $\hat{f}$ from both sizes of (29), we obtain

30 $$\begin{aligned} \Delta _{k+1} \le \Delta _k +\eta _k \left\langle \nabla f(w_k), x_{k}-w_k \right\rangle +\frac{L}{2} \eta _k^2 \Vert x_{k}-w_k\Vert ^2. \end{aligned}$$

By the optimality of $x_k$ in (25), we have

31 $$\begin{aligned} \left\langle \nabla f(w_k), x_k \right\rangle \le \left\langle \nabla f(w_k), \hat{w} \right\rangle . \end{aligned}$$

Assume from now on that $\Vert w_{k}-\hat{w} \Vert \ge \delta$. From (31) and the $(\varepsilon ,\delta )$-approximate convexity of *f* it follows that

32 $$\begin{aligned} \left\langle \nabla f(w_k), x_k -w_k \right\rangle\le & {} \left\langle \nabla f(w_k), \hat{w} -w_k \right\rangle \nonumber \\\le & {} f(\hat{w} )- f(w_k)+\frac{\varepsilon }{2}\Vert w_k-\hat{w} \Vert ^2 = -\Delta _k+\frac{\varepsilon }{2}\Vert w_k-\hat{w} \Vert ^2.\nonumber \\ \end{aligned}$$

Setting $z=\frac{-\nabla f(\hat{w} )}{\Vert \nabla f(\hat{w} )\Vert }$, we have $\Vert z\Vert =1$. By the strong convexity of *K* we obtain that

$$\begin{aligned} y_k := \frac{1}{2}(w_k+\hat{w} )+\frac{\gamma }{8} \Vert w_k-\hat{w} \Vert ^2 z \in K. \end{aligned}$$

Therefore, from the $(\varepsilon ,\delta )$-approximate convexity of *f* and the optimality of $\hat{w}$, we obtain

33 $$\begin{aligned} \Delta _k=f(w_k)-f(\hat{w} )\ge & {} \left\langle \nabla f(\hat{w} ), w_k-\hat{w} \right\rangle -\frac{\varepsilon }{2}\Vert w_k-\hat{w} \Vert ^2 \nonumber \\= & {} 2\left\langle \nabla f(\hat{w} ), \frac{w_k+\hat{w} }{2} -y_k \right\rangle + 2\left\langle \nabla f(\hat{w} ), y_k-\hat{w} \right\rangle \nonumber \\&-\frac{\varepsilon }{2}\Vert w_k-\hat{w} \Vert ^2\nonumber \\\ge & {} 2\left\langle \nabla f(\hat{w} ), \frac{w_k+\hat{w} }{2} -y_k \right\rangle - \frac{\varepsilon }{2}\Vert w_k-\hat{w} \Vert ^2\nonumber \\= & {} 2\left\langle \nabla f(\hat{w} ), \frac{\gamma }{8} \Vert w_k-\hat{w} \Vert ^2 \frac{ \nabla f(\hat{w} )}{\Vert \nabla f(\hat{w} )\Vert }\right\rangle -\frac{\varepsilon }{2}\Vert w_k-\hat{w} \Vert ^2 \nonumber \\= & {} \frac{\gamma }{4} \Vert \nabla f(\hat{w} )\Vert \Vert w_k-\hat{w} \Vert ^2-\frac{\varepsilon }{2}\Vert w_k-\hat{w} \Vert ^2\nonumber \\\ge & {} \left( \frac{\gamma \rho }{4}-\frac{\varepsilon }{2}\right) \Vert w_k-\hat{w} \Vert ^2 =\left( \nu +\frac{\varepsilon }{2}\right) \Vert w_k-\hat{w} \Vert ^2. \end{aligned}$$

Combining (33) with (32) we have

34 $$\begin{aligned} \left\langle \nabla f(w_k), x_k -w_k \right\rangle \le -\Delta _k+\frac{\varepsilon /2}{\nu +\varepsilon /2}\Delta _k =-\frac{\nu }{\nu +\varepsilon /2}\Delta _k. \end{aligned}$$

Setting $z_k=\frac{-\nabla f(w_k)}{\Vert \nabla f(w_k)\Vert }$, we have $\Vert z_k\Vert =1$. By the strong convexity of *K* we have that

$$\begin{aligned} y_k := \frac{1}{2}(w_k+x_k)+\frac{\gamma }{8} \Vert w_k-x_k\Vert ^2 z_k \in K. \end{aligned}$$

The optimality of $x_k$ in (25) yields that

35 $$\begin{aligned} \left\langle \nabla f(w_k), x_k -w_k \right\rangle\le & {} \left\langle \nabla f(w_k), y_k-w_k \right\rangle \nonumber \\= & {} \left\langle \nabla f(w_k), \frac{1}{2}(x_k-w_k)+\frac{\gamma }{8} \Vert w_k-x_k\Vert ^2 z_k \right\rangle \nonumber \\= & {} \frac{1}{2} \left\langle \nabla f(w_k),x_k-w_k \right\rangle \nonumber \\&+\frac{\gamma }{8} \Vert w_k-x_k\Vert ^2 \left\langle \nabla f(w_k), \frac{-\nabla f(w_k)}{\Vert \nabla f(w_k)\Vert }\right\rangle \nonumber \\= & {} \frac{1}{2} \left\langle \nabla f(w_k),x_k-w_k \right\rangle -\frac{\gamma }{8} \Vert w_k-x_k\Vert ^2 \Vert \nabla f(w_k)\Vert \nonumber \\\le & {} -\frac{1}{2}\frac{\nu }{\nu +\varepsilon /2}\Delta _k-\frac{\gamma }{8} \Vert w_k-x_k\Vert ^2 \Vert \nabla f(w_k)\Vert , \end{aligned}$$

where the last inequality follows from (34). Combining (30) with (35), we obtain that

$$\begin{aligned} \Delta _{k+1} \le \left( 1-\frac{\nu \eta _k}{2 \nu +\varepsilon } \right) \Delta _k - \frac{\eta _k}{2}\left( \frac{\gamma \Vert \nabla f(w_k)\Vert }{4} - L \eta _k \right) \Vert x_{k}-w_k\Vert ^2. \end{aligned}$$

$$\square $$

We are now in a position to establish the convergence results for the CGM.

#### Theorem 2.3

Let all the assumptions in Proposition 2.3 be satisfied. Assume also that $\Vert w_0-\hat{w}\Vert \ge \delta$ and the sequence $\{ w_k \}$ generated by the CGM satisfies $\Vert \nabla f(w_k)\Vert \ge \rho$ for all *k*. Let the number $\underline{\eta }$ and the sequence $\left\{ \eta _k \right\}$ be chosen such that

36 $$\begin{aligned} 0<\underline{\eta } \le \eta _k \le \min \left\{ 1, \frac{2 \nu +\varepsilon }{\nu }, \frac{\gamma \Vert \nabla f(w_k)\Vert }{4L} \right\} \quad \forall k. \end{aligned}$$

Then for every $k \in \mathbb {N}$, if $\Vert w_k-\hat{w}\Vert \ge \delta$ then

$$\begin{aligned} f(w_{k+1})-\hat{f} \le \theta \big ( f(w_{k})-\hat{f} \big ), \end{aligned}$$

where $\theta = 1- \frac{\nu \underline{\eta }}{2\nu +\varepsilon } \in (0,1)$. Moreover, for every *k*, if $\Vert w_i-\hat{w}\Vert \ge \delta , \, i=0,\ldots ,k$, then

$$\begin{aligned} \Vert w_k-\hat{w} \Vert ^2 \le \frac{\Delta _0}{\nu +\varepsilon /2} \theta ^k, \end{aligned}$$

Clearly, in the case $\delta = 0$, the first and the second claims of the theorem mean that the sequences $\left\{ f(w_k)\right\}$ and $\left\{ w_k\right\}$ converge linearly to $\hat{f}$ and $\hat{w}$, respectively. In the case $\delta > 0$ we also have linear convergence at least until the generated sequence enters the $\delta$-neighborhood of $\hat{w}$.

#### Proof

Take *k* with $\Vert w_k-\hat{w}\Vert \ge \delta$. From (36) we have

$$\begin{aligned} \frac{\gamma \Vert \nabla f(w_k)\Vert }{4} - L \eta _k \ge 0, \quad \text {and } \quad 1 \ge \frac{\nu \eta _k}{2 \nu +\varepsilon } \ge \frac{\nu \underline{\eta }}{2 \nu +\varepsilon } \quad \forall k. \end{aligned}$$

Therefore, it follows from (28) that, for all *k*, it holds

$$\begin{aligned} \Delta _{k+1} \le \left( 1- \frac{\nu \underline{\eta }}{2\nu +\varepsilon } \right) \Delta _k, \end{aligned}$$

which implies

37 $$\begin{aligned} f(w_{k+1})- \hat{f} \le \theta \left( f(w_{k})-\hat{f}\right) . \end{aligned}$$

In addition, if $\Vert w_i-\hat{w}\Vert \ge \delta , \, i=0,\ldots ,k$, then we have

$$\begin{aligned} \Delta _k \le \theta ^k \Delta _0. \end{aligned}$$

This and (33) imply

$$\begin{aligned} \Vert w_k-\hat{w} \Vert ^2 \le \frac{1}{\nu +\varepsilon /2} \Delta _k \le \frac{\Delta _0}{\nu +\varepsilon /2} \theta ^k. \end{aligned}$$

$$\square $$

## The Affine Optimal Control Problem

In this section we turn back to the control–affine linear-quadratic problem (1)–(3) and prove that the gradient projection methods considered in the previous section are applicable to the (high order) discretization of the problem recently developed in [21, 24]. (This also applies to the conditional gradient method, where the analysis is similar). We also provide error estimates regarding both the errors due to discretization and those due to truncation of the gradient projection iterations.

The first two subsections reproduce assumptions and results from [24] that are necessary for understanding the implementation of the GPM to the discretized version of problem (1)–(3). The next subsections prove the applicability of the abstract results obtained above, present details about the implementation of the gradient methods, and provide results of computational experiments.

### Notations and Assumptions

It will be convenient to introduce the space $H := (({\mathbb {R}}^2)^m)^N$ consisting of vectors $w = (w_0, \ldots , w_{N-1})$ with $w_i = (w_i^1, \ldots , w_i^m)$ and $w_i^j = (u_i^j,v_i^j) \in {\mathbb {R}}^2$. We regard this space as a Hilbert space with the scalar product

$$\begin{aligned} \langle w, \tilde{w} \rangle := \frac{1}{N} \sum _{i=0}^{N-1} \sum _{j=1}^m \langle w_i^j, \tilde{w}_i^j \rangle , \quad \langle w_i^j, \tilde{w}_i^j \rangle := u_i^j \tilde{u}_i^j + v_i^j \tilde{v}_i^j \end{aligned}$$

The scalar product is normalized by division by *N* since below *N* will be a “large” number and the sum will be a proxy for integration on a fixed interval [0, *T*] by using values on a mesh with size $h = T/N$. We also denote $|w_i| := \sqrt{\sum _{j=1}^m |w_i^j|^2}$, $|w_i^j|^2 := (|u_i^j|^2 + |v_i^j|^2)$. The $l_1$, $l_2$, and $l_\infty$ norms in *H* will be respectively

38 $$\begin{aligned} \Vert w \Vert _1 := \frac{1}{N} \sum _{i=0}^{N-1} | w_i |, \quad \Vert w \Vert _2 := \sqrt{ \frac{1}{N} \sum _{i=0}^{N-1} | w_i |^2}, \quad \Vert w \Vert _\infty = \max _i |w_i|. \end{aligned}$$

Clearly, the inequality $\Vert w\Vert _1 \le \Vert w\Vert _2 \le \Vert w\Vert _\infty$ holds for every $w \in H$.

As usual, $L_2([0,T];{\mathbb {R}}^m)$ denotes the Hilbert space of all measurable square-integrable functions $[0,T] \rightarrow {\mathbb {R}}^m$ with scalar product $\langle u_1, u_2 \rangle = \int _0^T \langle u_1(t), u_2(t) \rangle {\mathrm{\,d}}t$ and the corresponding norm is denoted again by $\Vert \cdot \Vert _2$.

We begin with some assumptions concerning the problem (1)–(3).

#### Assumption A1

The matrix functions *A*(*t*), *B*(*t*), *W*(*t*) and *S*(*t*), $t \in [0,T]$, have Lipschitz continuous first derivatives, *Q* and *W*(*t*) are symmetric. Moreover, the matrix $B(t)^{\top }S(t)$ is symmetric for all $t\in [0,T]$.

Denote by ${\mathcal {F}}$ the set of all admissible control–trajectory pairs (*u*, *x*), that is, all pairs of an admissible control *u* and the corresponding (absolutely continuous) solution *x* of (2). By a standard argument, problem (1)–(3) has a solution, $(\hat{x},\hat{u}) \in {\mathcal {F}}$, which from now on will be considered as fixed.

#### Assumption A2

$$\begin{aligned}&\frac{1}{2} z(T)^\top Qz(T)+q^\top z(T) \\&\quad +\int _0^T \left( \frac{1}{2}z(t)^\top W(t)z(t)+z(t)^\top S(t)v(t) \right) {\mathrm{\,d}}t \ge 0 \qquad \forall \, (z,v) \in {\mathcal {F}}- (\hat{x},\hat{u}). \end{aligned}$$

The first part of Assumption (A1) is standard, while the last requirement is demanding but known from the literature, usually expressed in terms of the Lie brackets of the involved controlled vector fields see e.g. [26]. It is certainly fulfilled in the case of single-input systems, $m = 1$. Assumption (A2) is a directional convexity assumption at $(\hat{x},\hat{u})$, which is somewhat weaker than the usual convexity assumption for the functional *J* in (1) regarded as a functional on the set of admissible controls (viewing *x* as a function of *u*).

The Pontryagin principle implies that there exists an absolutely continuous function $\hat{p} : [0,T] \rightarrow {\mathbb {R}}^n$ such that the triple $(\hat{x},\hat{u},\hat{p})$ satisfies the following system of generalized equations: for a.e. $t \in [0,T]$,

39 $$\begin{aligned}&0 = \dot{x}(t)-A(t)x(t)-B(t)u(t), x(0)=x_0, \end{aligned}$$

40 $$\begin{aligned}&0 = \dot{p}(t)+A(t)^\top p(t)+W(t)x(t)+S(t)u(t), \end{aligned}$$

41 $$\begin{aligned}&0 \in B(t)^\top p(t)+S(t)^\top x(t)+N_U(u(t)), \end{aligned}$$

42 $$\begin{aligned}&0 = p(T)-Qx(T)-q, \end{aligned}$$

where $N_U(u)$ is the normal cone to *U* at *u*. Following [10], we assume that the optimal control $\hat{u}$ is *strictly bang–bang*, with a finite number of switching times on [0, *T*], and that the so-called *switching function*,

$$\begin{aligned} \hat{\sigma }(t) := B(t)^\top \hat{p}(t)+S(t)^\top \hat{x}(t), \end{aligned}$$

exhibits a linear growth in a neighborhood of any zero.

#### Assumption A3

(strict bang–bang property)

There exist real numbers $\alpha ,\tau >0$ such that for all $j\in \{1,\dots ,m\}$ and $s\in [0,T]$ with $\hat{\sigma }^j(s)=0$ (the *j*-th component of $\hat{\sigma }$) we have

$$\begin{aligned} |\hat{\sigma }^j(t)| \ge \alpha |t-s| \quad \forall t \in [s-\tau ,s+\tau ]\cap [0,T]. \end{aligned}$$

Assumptions (A1)–(A3) will be standing in this section.

### High-Order Time-Discretization

In this subsection we recall the discretization scheme for problem (1)–(3) presented in [24], which has a higher accuracy than the Euler scheme without a substantial increase of the numerical complexity of the discretized problem. The approach uses second order truncated Volterra–Fliess series. The discretization scheme is described as follows.

For any natural number *N* denote $h = T/N$ and define the mesh $\{t_i\}_0^N$ with $t_i = i h$. Introducing the notations

$$\begin{aligned} A_i:= & {} A(t_i)+\frac{h}{2}\left( A(t_i)^2+ \dot{A}(t_i) \right) , \\ B_i:= & {} B(t_i)+h A(t_i)B(t_i), \\ C_i:= & {} - A(t_i)B(t_i)+\dot{B}(t_i), \end{aligned}$$

we replace the differential equation (2) with the discrete-time controlled dynamics

43 $$\begin{aligned} x_{i+1}= & {} x_i+h(A_i x_i + B_i u_i+h C_i v_i), \quad i=0,\ldots ,N-1, \quad x_0 \,\, \text{ given }, \end{aligned}$$

44 $$\begin{aligned}&w_i := (u_i,v_i) \in Z^m , \quad i=0,\ldots ,N-1, \end{aligned}$$

where $Z^m$ is the Cartesian product $\Pi _1^m Z$ and *Z* is the Aumann integral

$$\begin{aligned} Z:=\int _{0}^{1} \left( \begin{array}{c} 1\\ s\end{array}\right) \left[ -1, 1\right] ds. \end{aligned}$$

As pointed out in [21], the set *Z* can be easily represented in the more convenient way as

45 $$\begin{aligned} Z=\left\{ \left( \alpha , \beta \right) : \alpha \in [-1,1], \beta \in [\varphi _1(\alpha ), \varphi _2(\alpha )] \right\} , \end{aligned}$$

where $$\varphi _1(\alpha ):=\frac{1}{4}(-1+2\alpha +\alpha ^2) $$ and $$\varphi _2(\alpha ):=\frac{1}{4}(1+2\alpha -\alpha ^2)$$.

**Fig. 1:**
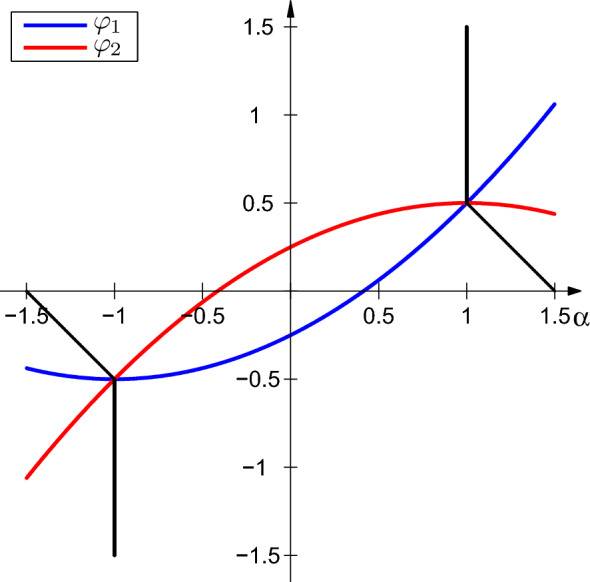
The set *Z* as the area between the two parabolas $\varphi _1$ (lower) and $\varphi _2$ (upper)

For the subsequent analysis it will be important that the set $Z \subset {\mathbb {R}}^2$ is strongly convex. This is evident from Fig. 1, but the calculation of a modulus $\gamma$ is cumbersome and we skip the details. In this calculation we use Theorem 1 in [28] (expressing $\gamma$ by the Lipschitz constant of the mapping that maps a unit vector to that point on the boundary of *Z* at which this vector is normal to *Z*) and the explicit formula for the normal cone to *Z* given in [21, Sect. 4]. The number $\gamma = 1/\sqrt{32}$ turns out to be a modulus of strong convexity of *Z*.

We introduce the discrete-time counterpart of the objective functional *J* in (1): for $x=\left( x_0,\ldots ,x_N \right)$, $w = (w_0, \ldots , w_{N-1}) = \left( (u_0,v_0), \ldots , (u_{N-1}, v_{N-1}) \right)$,

46 $$\begin{aligned} J^h(x,w):= & {} \frac{1}{2} x^\top _N\left( Qx_N+q \right) + \frac{h}{2} \sum _{i=0}^{N-1} \big ( x^\top _i W(t_i)\left( x_i+hA(t_i)x_i\right) + \frac{h}{2} x^\top _i \dot{W}(t_i) x_i\big )\nonumber \\&+\, h \sum _{i=0}^{N-1} \Big ( hx_i^\top W(t_i)B(t_i)(u_i-v_i)+x_i^\top \big ( S(t_i)u_i+h \dot{S}(t_i)v_i\big )\nonumber \\&+\, h\left( A(t_i)x_i\right) ^\top S(t_i)v_i+\frac{h}{2} u_i^\top B(t_i)^\top S(t_i)u_i \Big ). \end{aligned}$$

Then we consider the problem of minimization of the functional $J^h$ defined in (46) subject to the constraints (43)–(44). The set of admissible discrete controls in this problem is denoted by $K \subset H$, that is,

$$\begin{aligned} K := \{ (w_0, \ldots , w_{N-1}) \in {\mathbb {R}}^{2m \times N} : \,w_i = (u_i,v_i) \in Z^m \}. \end{aligned}$$

We also introduce the discrete adjoint equation (see formula (3.11) in [24])

47 $$\begin{aligned} p_i= & {} \left( I + h A_i^{\top }\right) p_{i+1} + h \left( S(t_i)u_i + h \dot{S}(t_i)v_i+ h A(t_i)^{\top }S(t_i)v_i \right) \nonumber \\&+\, h \left( W(t_i) + \frac{h}{2} W(t_i) A(t_i)+ \frac{h}{2} A(t_i)^{\top }W(t_i) + \frac{h}{2} \dot{W}(t_i)\right) x_i\nonumber \\&+\, h^2 W(t_i)B(t_i)( u_i - v_i ), \end{aligned}$$

$i = N-1, \ldots , 0$, with the end condition

48 $$\begin{aligned} p_N = Q^{\top }x_N+q. \end{aligned}$$

Section 3.3 in [24] presents a construction which for every sequence $w =(w_0, \ldots w_{N-1}) \in K$ defines an admissible control $u = \Phi ^h(w)$ in problem (1)–(3), with values $\pm 1$ and with at most two switches in every interval $[t_i,t_{i+1}]$ of each of its components. We do not reproduce this construction here, only mentioning that it requires only a few calculations (to define the switching points), and the restriction of any component $j=1,\ldots ,m$ of $u(t) = \Phi ^h(w)(t)$ to $[t_i,t_{i+1}]$ depends only on $w_i^j$. Moreover, the following equalities hold (see (3.14) in [24]): for every $w = ((u_0,v_0), \ldots , (u_{N-1},v_{N-1}))$

49 $$\begin{aligned} \int _{t_i}^{t_{i+1}} \Phi ^h(w)(s) {\mathrm{\,d}}s = h u_i, \quad \int _{t_i}^{t_{i+1}} (s-t_i) \Phi ^h(w)(s) {\mathrm{\,d}}s = h^2 v_i, \quad i=0, \ldots , N-1. \end{aligned}$$

In addition, the function $\Phi ^h$ has the important property that there exists a constant $\tilde{c}$ independent of *N* such that for every *i*, *j* and $w_i^j, \tilde{w}_i^j \in Z$

$$\begin{aligned} \int _{t_i}^{t_{i+1}} \left| [\Phi ^h (w) - \Phi ^h(\tilde{w})]^j\right| {\mathrm{\,d}}t \le \frac{\tilde{c}}{N} |w_i^j - \tilde{w}_i^j|. \end{aligned}$$

Clearly, this implies

50 $$\begin{aligned} \Vert \Phi ^h(w) - \Phi ^h(\tilde{w}) \Vert _1 \le \tilde{c} \Vert w - \tilde{w}\Vert _1 \qquad \forall w, \tilde{w} \in K. \end{aligned}$$

Below we will use the metric

$$\begin{aligned} d^{\#} (u_1,u_2) = \text{ meas } \left\{ t\in [0,1]: u_1(t)\ne u_2(t) \right\} \end{aligned}$$

in the set of admissible controls in problem (1)–(3).

The following theorem is extracted from Theorem 3.1 in [24].

#### Theorem 3.1

Let Assumption (A1) be fulfilled. Let $(\hat{x}, \hat{u})$ be a solution of problem (1)–(3) for which assumptions (A2) and (A3) are fulfilled, and let $\hat{p}$ the corresponding solution of the adjoint equation (40) with end-condition (42). Then for every natural number *N* the problem of minimization of (46) under constrains (43)–(44) has a solution $(\hat{x}^N,\hat{w}^N) = \{(\hat{x}_i^N,\hat{w}_i^N)\}$ and for every such solution and the corresponding discrete adjoint sequence $(\hat{p}_0^N, \ldots , \hat{p}_N^N)$ solving (47), (48), the following error estimate holds:

51 $$\begin{aligned} \max _{i=0, \ldots , N} \left( |\hat{x}_i^N - \hat{x}(t_i)\right| + |\hat{p}_i^N - \hat{p}(t_i)|) \,+ \, d^{\#} \left( \Phi ^h(\hat{w}^N), \hat{u} \right) \, \le \, c\, h^{2}, \end{aligned}$$

where *c* is independent of *N*.

We mention that the above discretization scheme is meaningful even without assuming (A2) and (A3). These assumptions are only needed for the error estimate in Theorem 3.1.

### Applicability of the Results About Gradient-Type Methods

First of all, we reformulate the problem of minimization of (46) under the constraints (43)–(44) as a minimization problem on the set

52 $$\begin{aligned} K := \prod _0^{N-1} Z^m \subset H, \end{aligned}$$

namely,

53 $$\begin{aligned} \mathop {{\mathrm{minimize}}}\limits _{w \in K} \left\{ f^h(w) := J^h(x^h[w],w) \right\} , \end{aligned}$$

where $x^h[w]$ is the solution of the discrete-time equation (43) for $w = (w_0, \ldots , w_{N-1}) \in K$, $w_i = ((u_i^1, v_i^1), \ldots (u_i^m, v_i^m)) \in Z^m$, with the given initial condition $x_0$.

In this subsection we prove that the assumptions needed for applicability of the results in Sect. 2 to the above problem are fulfilled.

Let us denote by *f* the objective functional in problem (1)–(3), regarded as a function of the control, namely, $f(u) := J(x[u],u)$, where *x*[*u*] is the solution of (2) corresponding to $u \in L_2([0,T];{\mathbb {R}}^m)$. It is well known that the functional $f : L_2([0,T];{\mathbb {R}}^m) \rightarrow {\mathbb {R}}$ is Fréchet differentiable at any *u* and its derivative has the functional representation

54 $$\begin{aligned} \nabla f(u)(t) = B(t)^\top p(t)+S(t)^\top x(t), \end{aligned}$$

where *x* and *p* are the solutions of (39), (40), (42) corresponding to *u*. Similarly, the function $f^h : H \rightarrow {\mathbb {R}}$ is Fréchet differentiable, and its derivative has the representation (see the second term in the right hand side of (3.12) in [24])

55 $$\begin{aligned} \nabla _{w_i} f^h (w)= & {} \left( \begin{array}{c} \nabla _{u_i} f^h (w) \\ \nabla _{v_i} f^h (w) \end{array} \right) \nonumber \\= & {} \left( \begin{array}{c} B^{\top }_i p_{i+1} + S(t_i)^{\top }x_i+ h B(t_i)^{\top }W(t_i)x_i + h B(t_i)^{\top } S(t_i) u_i\\ h \left( C_i^{\top }p_{i+1} - B(t_i)^{\top } W(t_i)x_i + \left( S(t_i)^{\top }A(t_i) + \dot{S}(t_i)^\top \right) x_i \right) \end{array} \right) . \nonumber \\ \end{aligned}$$

We mention that Assumption (A2) implies that *f* is convex at $\hat{u}$, hence

56 $$\begin{aligned} \langle \nabla f(u) - \nabla f(\hat{u}), u - \hat{u} \rangle \ge 0 \qquad \text{ for } \text{ all } \text{ admissible } \text{ controls } \text{ u }. \end{aligned}$$

In contrast, $f^h$ does not need to be convex.

Next, we present five technical lemmas which are needed in the proof of the main result in this section—Proposition 3.1. In the proofs, $c_1, c_2, \ldots$ denote non-negative constants that may depend on the data of the problem (1)–(3) (and their derivatives) but are independent of *N*. These constants may have different values in different proofs.

#### Lemma 3.1

There exist constants $c'$ and $c''$ independent of *h*, such that for every $w', w'' \in K$ and $\Delta w \in K-K$

$$\begin{aligned} | \langle \nabla f^h(w') - \nabla f^h(w''), \Delta w \rangle |\le & {} c' \Vert w' - w'' \Vert _1 \, \Vert \Delta w \Vert _1 + c'' h^2 \sum _{i=1}^{N-1} | u_i' - u_i''| \, |\Delta u_i| \\\le & {} c' \Vert w'-w''\Vert _1 \, \Vert \Delta w\Vert _1 + c''h \Vert w'-w''\Vert _2 \, \Vert \Delta w\Vert _2, \end{aligned}$$

where $u_i'$, $u_i''$, $\Delta u_i$ are the first coordinates of the components $w_i'$, $w_i''$, $\Delta w_i$ of the elements $w'$, $w''$ and $\Delta w$, respectively.

#### Proof

Considering the discrete equation (43), it is a standard procedure to obtain the following estimate for the solutions $x'$ and $x''$ corresponding to $w'$ and $w''$:

57 $$\begin{aligned} \Vert x' - x'' \Vert _\infty \le c_1 \Vert w'-w'' \Vert _1. \end{aligned}$$

Similarly, also using the last estimation, we obtain from (47), (48) that

58 $$\begin{aligned} \Vert p' - p'' \Vert _\infty \le c_2 \Vert w'-w'' \Vert _1. \end{aligned}$$

Then using the explicit representation (55) we obtain that

$$\begin{aligned} | \langle \nabla f^h(w') - \nabla f^h(w''), \Delta w \rangle |\le & {} c_1 \left( \Vert x' - x'' \Vert _\infty + \Vert p' - p'' \Vert _\infty \right) \Vert \Delta w\Vert _1\\&+\, c_2 h^2 \sum _{i=1}^{N-1} | u_i' - u_i''| \, |\Delta u_i|, \end{aligned}$$

which together with (57) and (58) implies the firts inequality in the lemma. The second one follows by application of the Cauchy–Schwarz inequality and the definition of the norms. $\square$

#### Lemma 3.2

There exists a number $c^*$ such that for every natural number *N*, for every $\bar{w} \in K$ and for every $\Delta \in L_2([0,T]; {\mathbb {R}}^m)$

$$\begin{aligned} \left| \langle \nabla f(\Phi ^h(\bar{w})), \Delta \rangle - T \left\langle \nabla f^h(\bar{w}), w(\Delta ) \right\rangle \right| \le c^* h^2 \Vert \Delta \Vert _1, \end{aligned}$$

where $w(\Delta ) :=\{(u_i,v_i)\}_0^{N-1}$ is defined as

$$\begin{aligned} u_i = \frac{1}{h} \int _{t_i}^{t_{i+1}} \Delta (t) {\mathrm{\,d}}t, \quad v_i = \frac{1}{h^2} \int _{t_i}^{t_{i+1}} (t-t_i) \Delta (t) {\mathrm{\,d}}t. \end{aligned}$$

#### Proof

Denote by $\bar{x}$ and $\bar{p}$ the solutions of (39) and (40), (42), corresponding to the control function $\bar{u} := \Phi ^h(\bar{w})$. Similarly we denote by $\{\bar{x}_i\}$ and $\{ \bar{p}_i\}$ the solutions of (43) and (47), (48), corresponding to $\bar{w}$. The results in points 2 and 3 in [24, Sect. 4] (see (4.5) there) imply that for $t \in [t_i,t_{i+1}]$

$$\begin{aligned} B^{\top }(t) \bar{p}(t)+ S(t)^\top \bar{x}(t)= & {} (B_i+ (t-t_i)C_i)^{\top } \bar{p}_{i+1} \nonumber \\&+\, B(t_i)^{\top }\left( (t_{i+1}-t) W(t_i) \bar{x}_i + S(t_i)\int _{t_i}^{t_{i+1}} \! \bar{u}(s) {\mathrm{\,d}}s \right) \nonumber \\&+ \, S(t_i)^{\top } \left( I + (t-t_i)A(t_i) \right) \bar{x}_i\nonumber \\&+ \dot{S}(t_i)^{\top }(t-t_i) \bar{x}_i +O(t;h^2), \end{aligned}$$

where $O(t;h^2)$ is measurable in *t* and $|O(t;h^2)| \le c_1 h^2$ for a.e. *t*. Using this expression and (54) we obtain the following equality:

$$\begin{aligned} \langle \nabla f(\Phi ^h(\bar{w})), \Delta \rangle= & {} \int _0^T \langle B^{\top }(t) \bar{p}(t)+ S(t)^\top \bar{x}(t), \Delta (t) \rangle {\mathrm{\,d}}t \\= & {} \sum _{i=0}^{N-1} \int _{t_i}^{t_{i+1}} \Big \langle ( B_i+ (t-t_i)C_i)^{\top } \bar{p}_{i+1}\\&+\, B(t_i)^{\top }\Big ( (t_{i+1}-t) W(t_i) \bar{x}_i + S(t_i)\int _{t_i}^{t_{i+1}} \! \bar{u}(s) {\mathrm{\,d}}s \Big )\nonumber \\&+ \, S(t_i)^{\top } \left( I + (t-t_i)A(t_i) \right) \bar{x}_i \\&+\, \dot{S}(t_i)^{\top }(t-t_i) \bar{x}_i +O(t;h^2), \Delta (t) \Big \rangle {\mathrm{\,d}}t. \end{aligned}$$

Using the expressions (55) we obtain, after a simple rearrangement of terms, that

$$\begin{aligned}&\langle \nabla f(\Phi ^h(\bar{w})), \Delta \rangle \\&\quad = \sum _{i=0}^{N-1} \left[ \Big \langle \nabla _{u_i} f^h(\bar{w}), \int _{t_i}^{t_{i+1}} \Delta (t) {\mathrm{\,d}}t \Big \rangle + \Big \langle \frac{1}{h} \nabla _{v_i} f^h(\bar{w}), \int _{t_i}^{t_{i+1}} (t-t_i) \Delta (t) {\mathrm{\,d}}t \Big \rangle \right] \\&\qquad +\, \int _0^T \langle O(t;h^2), \Delta (t) \rangle {\mathrm{\,d}}t \\&\quad = \sum _{i=0}^{N-1} \left[ \Big \langle \nabla _{u_i} f^h(\bar{w}), h u_i \Big \rangle + \Big \langle \nabla _{v_i} f^h(\bar{w}), h v_i \Big \rangle \right] + \int _0^T \langle O(t;h^2), \Delta (t) \rangle {\mathrm{\,d}}t \\&\quad = h \sum _{i=0}^{N-1} \Big \langle \nabla _{w_i} f^h(\bar{w}), w_i(\Delta ) \Big \rangle + \int _0^T \langle O(t;h^2), \Delta (t) \rangle {\mathrm{\,d}}t \\&\quad = T \langle \nabla f^h(\bar{w}), w(\Delta ) \rangle + \int _0^T \langle O(t;h^2), \Delta (t) \rangle {\mathrm{\,d}}t. \end{aligned}$$

Then the estimation $|O(t;h^2)| \le c_1 h^2$ completes the proof. $\square$

#### Lemma 3.3

The function $f^h$ defined in (53) is *L*-smooth on *K* with the Lipschitz constant of its derivative being independent of *N*:

$$\begin{aligned} \Vert \nabla f^h(w') - \nabla f^h(w'') \Vert _2 \le L \Vert w' - w'' \Vert _2. \end{aligned}$$

#### Proof

The Fréchet differentiability of $f^h$ was established in [24]), together with the representation (55) of its derivative. The Lipschitz continuity on *K* follows from this representation, together with (57) and (58) (the notations are as in the proof of Lemma 3.1). $\square$

We remind that $\hat{w}^N \in K$ denoted in Theorem 3.1 an optimal control sequence in the discrete problem (53). Further it will be convenient to skip the superscript *N* in this notation.

#### Lemma 3.4

There exist numbers $N_0$ and $\delta _1$ such that for every $N \ge N_0$

$$\begin{aligned} \langle \nabla f^h(\hat{w}), w - \hat{w} \rangle \ge \frac{\alpha \gamma }{64} h \Vert w-\hat{w}\Vert _2^2 \quad \text{ for } \text{ every } w \in K \text{ with } \Vert w-\hat{w}\Vert _2 \ge \delta _1 \sqrt{h} \end{aligned}$$

($\alpha$ is the number from Assumption (A3) and $\gamma \ge 1/\sqrt{32}$ is a modulus of strong convexity of *Z*).

#### Proof

The following expression is obtained in [24] (see formula (4.5) there, applied for $t=t_{i+1}$):

$$\begin{aligned} B(t_{i+1})^\top \hat{p}_{i+1}^N + S(t_{i+1})^\top \hat{x}_{i+1}^N= & {} (B_i + h C_i)^\top \hat{p}_{i+1}^N + B(t_i)^\top S(t_i) \int _{t_i}^{t_{i+1}} u(s) {\mathrm{\,d}}s \\&+\, S(t_i)^\top (I + h A(t_i)) \hat{x}_i^N + h \dot{S}(t_i)^\top \hat{x}_i^N + O(h^2), \end{aligned}$$

where $u = \Phi ^h(\hat{w})$. Comparing this with the expression (55) we see that

$$\begin{aligned} B(t_{i+1})^\top \hat{p}_{i+1}^N + S(t_{i+1})^\top \hat{x}_{i+1}^N = \nabla _{\!u_i} f^h(\hat{w}) + \nabla _{\!v_i} f^h(\hat{w}) + O(h^2). \end{aligned}$$

Then using Theorem 3.1 we obtain that

$$\begin{aligned}&|\hat{\sigma }(t_{i+1}) - \nabla _{u_i} f^h(\hat{w}) - \nabla _{v_i} f^h(\hat{w}) | \\&\quad \le \Big |B(t_{i+1})^\top \hat{p}(t_{i+1}) + S(t_{i+1})^\top \hat{x}(t_{i+1}) - B(t_{i+1})^\top \hat{p}_{i+1}^N - S(t_{i+1})^\top \hat{x}_{i+1}^N \Big | \le \bar{c} h^2, \end{aligned}$$

where $\bar{c}$ is an appropriate constant. Written for the *j*th components of the vectors in the left-hand side, the inequality becomes

59 $$\begin{aligned} |\hat{\sigma }^j(t_{i+1}) - \nabla _{\!u_i^j} f^h(\hat{w}) - \nabla _{\!v_i^j} f^h(\hat{w}) | \le \bar{c} h^2, \quad j = 1, \ldots , m. \end{aligned}$$

Assumption (A3) implies that there exist a natural number *r* and a real number $\tau _0 \in (0,\tau )$ such that every component $\hat{\sigma }^j$ of $\hat{\sigma }$ has at most *r* zeros in [0, *T*], and $|\hat{\sigma }^j(t)| \ge \alpha \tau '$ every $\tau ' \in (0,\tau _0]$ and for every *t* which does not belong to a $\tau '$-neighborhood of a zero of $\hat{\sigma }^j$.

Now, let us define $\delta _1 := M \sqrt{2mr/T}$, where *M* is the diameter of the set *Z* (which is $\sqrt{5}$). Moreover, define the natural number $N_0$ as bigger than $4 \bar{c} T/\alpha$, so that $\bar{c}h \le \alpha /4$.

Let $w = (w_0, \ldots , w_{N-1})$ with $w_i =(w_i^1, \ldots , w_i^m)$ and $w_i^j = (u_i^j,v_i^j) \in Z$ be arbitrarily chosen. Due to the $\gamma$-strong convexity of *Z* we have that

$$\begin{aligned} y_i^j := \frac{1}{2} (w_i^j - \hat{w}_i^j) + \frac{\gamma }{8} |w_i^j - \hat{w}_i^j|^2 \zeta _i^j \in Z \end{aligned}$$

for every $\zeta _i^j \in {\mathbb {R}}^2$ with $|\zeta _i^j| \le 1$. With the choice $\zeta _i^j = - \nabla _{\!w_i^j} f^h(\hat{w})/|\nabla _{w_i^j} f^h(\hat{w})|$ (whenever the denominator is non-zero) we obtain exactly in the same way as in the proof of Proposition 2.1 that

$$\begin{aligned} \langle \nabla f^h(\hat{w}), w - \hat{w} \rangle= & {} \frac{1}{N} \sum _{i=0}^{N-1} \sum _{j=1}^m \big \langle \nabla _{\!w_i^j} f^h(\hat{w}) , w_i^j - \hat{w}_i^j \big \rangle \\= & {} 2 \langle \nabla f^h(\hat{w}), y - \hat{w} \rangle - \frac{\gamma }{4 N} \sum _{i=0}^{N-1} \sum _{j=1}^m |w_i^j - \hat{w}_i^j|^2 \, \big \langle \nabla _{\!w_i^j} f^h(\hat{w}), \zeta _i^j \big \rangle \\\ge & {} \frac{\gamma }{4 N} \sum _{i=0}^{N-1} \sum _{j=1}^m |w_i^j - \hat{w}_i^j|^2 \, \big |\nabla _{\!w_i^j} f^h(\hat{w})\big |. \end{aligned}$$

Denote by $\Omega ^j$ the set of all indexes *i* such that $|\nabla _{\!w_i^j} f^h(\hat{w})|< \alpha h/8$. Then

60 $$\begin{aligned} \langle \nabla f^h(\hat{w}), w - \hat{w} \rangle\ge & {} \frac{\gamma }{4 N} \sum _{j=1}^m \sum _{i \not \in \Omega ^j} \frac{\alpha h}{8} |w_i^j - \hat{w}_i^j|^2 \nonumber \\= & {} \frac{\gamma \alpha h}{32 N} \left[ \sum _{j=1}^m \sum _{i=0}^{N-1} |w_i^j - \hat{w}_i^j|^2 - \sum _{j=1}^m \sum _{i \in \Omega ^j} |w_i^j - \hat{w}_i^j|^2 \right] \nonumber \\= & {} \frac{\gamma \alpha h}{32 } \left[ \Vert w - \hat{w}\Vert _2^2 - \frac{1}{N}\sum _{j=1}^m \sum _{i \in \Omega ^j} |w_i^j - \hat{w}_i^j|^2 \right] . \end{aligned}$$

Consider an arbitrary $i \in \Omega ^j$. Since $|\nabla _{\!w_i^j}f^h(\hat{w})| < \alpha h/8$, according to (59) we have

$$\begin{aligned} |\hat{\sigma }^j(t_{i+1})| < \frac{\alpha h}{4} + \bar{c} h^2 \le \frac{\alpha h}{2}, \end{aligned}$$

where we also use that $\bar{c} h \le \alpha /4$. Then $t_{i+1}$ belongs to the *h* / 2-neighborhood of some zero of $\hat{\sigma }^j$ (see the paragraph after (59)). Then no other point $t_k \not = t_{i+1}$ belongs to this neighborhood. Since $\hat{\sigma }^j$ has at most *r* zeros, the set $\Omega ^j$ consists of at most *r* points. Then continuing (60) we obtain

$$\begin{aligned} \langle \nabla f^h(\hat{w}), w - \hat{w} \rangle \ge \frac{\gamma \alpha h}{32} \left[ \Vert w - \hat{w}\Vert _2^2 - \frac{1}{N} rm M^2 \right] \end{aligned}$$

Now assume that $\Vert w - \hat{w}\Vert _2 \ge \delta _1 \sqrt{h} = M \sqrt{2mr/T}\sqrt{h}$. Then

$$\begin{aligned} \langle \nabla f^h(\hat{w}), w - \hat{w} \rangle \ge \frac{\gamma \alpha h}{32} \Vert w - \hat{w}\Vert _2^2 \left[ 1- \frac{1}{ (\delta _1)^2 h} \frac{rm M^2}{N} \right] = \frac{\gamma \alpha h}{64} \Vert w - \hat{w}\Vert _2^2. \end{aligned}$$

The proof is complete. $\square$

#### Lemma 3.5

There exists a constant $\nu _1 > 0$ such that

61 $$\begin{aligned} \langle \nabla f^h(w) - \nabla f^h(\hat{w}), w - \hat{w} \rangle \ge - \nu _1 h^2 (\Vert w-\hat{w}\Vert _2 + h) \quad \text{ for } \text{ every } w \in K. \end{aligned}$$

#### Proof

As before, let $\hat{u}$ be the optimal control in the continuous-time problem (1)–(3). Denote

$$\begin{aligned} \tilde{w}_i = \left( \frac{1}{h} \int _{t_i}^{t_{i+1}} \hat{u}(t) {\mathrm{\,d}}t, \frac{1}{h^2} \int _{t_i}^{t_{i+1}} (t - t_i) \hat{u}(t) {\mathrm{\,d}}t\right) . \end{aligned}$$

Denote $\mu _i := \text{ meas }\{ t \in [t_i,t_{i+1}] : \,\Phi ^h(\hat{w})(t) \not = \hat{u}(t) \}$. According to Theorem 3.1, $\sum _{i=0}^{N-1} \mu _i \le c h^2$. Due to (49), we have

$$\begin{aligned} |\tilde{w}_i - \hat{w}_i| \le \frac{c_1}{h} \mu _i, \end{aligned}$$

hence

62 $$\begin{aligned} \Vert \tilde{w} - \hat{w}\Vert _1 = \frac{1}{N} \sum _{i=0}^{N-1} |\tilde{w}_i - \hat{w}_i| \le \frac{h}{T} \sum _{i=0}^{N-1} \frac{c_1}{h} \mu _i \le c_2 h^2. \end{aligned}$$

Moreover,

63 $$\begin{aligned} \Vert \tilde{w} - \hat{w}\Vert _2 \le \sqrt{\Vert \tilde{w} - \hat{w}\Vert _\infty \, \Vert \tilde{w} - \hat{w}\Vert _1} \le c_3 h. \end{aligned}$$

Now we denote the left-hand side of (61) by *D* and represent $D = D_1 + D_2 + D_3$, where

$$\begin{aligned} D_1:= & {} \langle \nabla f^h(\tilde{w}) - \nabla f^h(\hat{w}), w - \hat{w} \rangle , \\ D_2:= & {} \langle \nabla f^h(w) - \nabla f^h(\tilde{w}), w - \tilde{w} \rangle , \\ D_3:= & {} \langle \nabla f^h(w) - \nabla f^h(\tilde{w}), \tilde{w} - \hat{w} \rangle . \end{aligned}$$

We shall estimate each of these terms separately. From Lemma 3.1 we obtain

$$\begin{aligned} D_1\ge & {} - c' \Vert \tilde{w}- \hat{w}\Vert _1 \, \Vert w - \hat{w}\Vert _1 - c''h \Vert \tilde{w}- \hat{w}\Vert _2 \, \Vert w - \hat{w}\Vert _2 \\\ge & {} - c' c_2 h^2 \Vert w - \hat{w}\Vert _1 - c'' c_3 h^2 \Vert w - \hat{w}\Vert _2 \ge - c_4h^2 \Vert w - \hat{w}\Vert _2. \end{aligned}$$

In order to estimate $D_2$ we use Lemma 3.2 and the definition of $\tilde{w}$:

$$\begin{aligned} D_2 \ge \frac{1}{T} \big \langle \nabla f(\Phi ^h(w)) - \nabla f(\hat{u}), \, \Phi ^h(w) - \hat{u} \big \rangle - \frac{2}{T} c^* h^2 \Vert \Phi ^h(w) - \hat{u}\Vert _1. \end{aligned}$$

The first term in the right-hand side is non-negative due to (56). Hence, using also (50), we obtain that

$$\begin{aligned} D_2\ge & {} - c_5 h^2 \Vert \Phi ^h(w) - \hat{u}\Vert _1 \ge - c_6 h^2 \Vert w-\tilde{w}\Vert _1 \ge -c_6 (\Vert w-\hat{w}\Vert _1 + \Vert \hat{w} - \tilde{w} \Vert _1)\\\ge & {} -c_6 (\Vert w-\hat{w}\Vert _1 + c_2 h^2). \end{aligned}$$

For estimating $D_3$ we use again Lemma 3.1, (62) and (63) :

$$\begin{aligned} D_3\ge & {} -c' \Vert w - \tilde{w}\Vert _1 \, \Vert \tilde{w} - \hat{w} \Vert _1 -c'' h \Vert w - \tilde{w}\Vert _2 \, \Vert \tilde{w} - \hat{w} \Vert _2 \\\ge & {} - c_7 h^2 \Vert w-\tilde{w}\Vert _1 - c_{8} h^2 \Vert w-\tilde{w}\Vert _2 \ge - c_{9} h^2 (\Vert w-\hat{w}\Vert _2 + \Vert \hat{w}-\tilde{w}\Vert _2) \\\ge & {} - c_{10} h^2 (\Vert w-\hat{w}\Vert _2 + h). \end{aligned}$$

Combining the estimations for $D_1$, $D_2$ and $D_3$ we obtain (61). $\square$

#### Proposition 3.1

On the assumptions (A1)–(A3), the function $f^h$ is *L*-smooth on *K* and there exist numbers $N_0$, $\nu _0 > 0$ and $\delta _0$ such that for every $N \ge N_0$ condition (7) in Proposition 2.1 (hence, also the assumptions in Proposition 2.2 and Theorems 2.1 and 2.2) is fulfilled for problem (53) with $\nu = \nu _0h$ and $\delta = \delta _0 \sqrt{h}$.

#### Proof

The *L*-smoothness of $f^h$ on *K* was proved in Lemma 3.3. Now, take an arbitrary $w \in K$ and consider

$$\begin{aligned} \left\langle \nabla f(w), w - \hat{w} \right\rangle = \langle \nabla f^h(\hat{w}), w - \hat{w} \rangle + \langle \nabla f^h(w) - \nabla f^h(\hat{w}), w - \hat{w} \rangle . \end{aligned}$$

Using Lemmas 3.4 and 3.5 we estimate

$$\begin{aligned} \left\langle \nabla f(w), w - \hat{w} \right\rangle \ge \frac{\alpha \gamma }{64} h \Vert w-\hat{w}\Vert _2^2 - \nu _1 h^2 (\Vert w-\hat{w}\Vert _2 + h) \end{aligned}$$

for every $w \in K$ with $\Vert w-\hat{w}\Vert _2 \ge \delta _1 \sqrt{h}$. Then for such *w* it holds that

$$\begin{aligned} \left\langle \nabla f(w), w - \hat{w} \right\rangle\ge & {} \frac{\alpha \gamma }{64} h \Vert w-\hat{w}\Vert _2^2 - \frac{\nu _1 h^2}{\delta _1 \sqrt{h}} \Vert w-\hat{w}\Vert _2^2 - \frac{\nu _1 h^3}{(\delta _1)^2 h} \Vert w-\hat{w}\Vert _2^2 \\= & {} h \Vert w-\hat{w}\Vert _2^2 \,\left( \frac{\alpha \gamma }{64} - \frac{\nu _1 h^{1/2}}{\delta _1} - \frac{\nu _1 h}{(\delta _1)^2 } \right) . \end{aligned}$$

Then the claim of the proposition holds for all sufficiently small *h*. $\square$

Let us interpret the above proposition in view of Theorem 2.1 for convergence of the gradient projection method (GPM) applied to the discrete problem (52) and (53). The linear rate of convergence, $\mu$, as estimated in this theorem, may approach 1 when $\nu$ approaches zero. In the same time, Proposition 3.1 estimates $\nu$ as proportional to *h*. Thus, although the convergence is linear, its rate, $\mu$, may be close to one. Even more, this rate of convergence is valid only until an accuracy $\delta$ is achieved (see Theorem 2.1). The number $\delta$ in Proposition 3.1 is estimated as proportional to $\sqrt{h}$. Thus the convergence of the GPM does not seem to be consistent with the $O(h^2)$-approximation that the discretization method provides. On the other hand, the fact that the GPM is proved to converge (even linearly, in the sense of Theorem 2.1) is remarkable. Indeed, if the Euler discretization scheme is applied to the original problem (1)–(3) (as in most of the literature), the resulting discrete-time problem may fail to be convex, and no results about the rate of convergence of the GPM are available in the literature, to the authors’ knowledge.

We do not present the convergence analysis of the CGM for problem (52) and (53), which is rather similar.

### Implementation of the Gradient Methods

Now, we shall describe the implementation of the GPM and the CGM to the specific mathematical programming problem defined by (53) and (52).

The two key points in the implementation of the gradient methods are: (i) calculation of the gradient $\nabla f^h(w)$; calculation of projections on *K* (for the GPM) or solving a linear optimization problem on *K* (for the CGM). We do not discuss here the issue of the choice of the step sizes $\lambda _k$, for which numerous possibilities are known from the literature.

**1. Calculation of**$\nabla f^h(w)$ Since $f^h$ represents the objective function of a discrete-time optimal control problem as a function of the control variables (the state being implicitly regarded as a function of the control), we employ the well known in control theory way for calculating its gradient: $\nabla f^h(w)$ is the derivative of the Hamiltonian with respect to the control, evaluated at the current control–trajectory pair, together with the corresponding solution of the adjoint equation. The explicit formula is given in (55), reproducing [24, Sect. 3.2].

**2. Calculation of the projection on ***K*

The set *K* is a product of $m\times N$ copies of the strongly convex set *Z*, thus the projection of a vector $w \in H$ onto *K* is represented by projections onto *Z* of the two-dimensional components of *w*. Thus we have to only calculate projections, $P_Z(u,v)$ on *Z*, where $(u,v)^\top \in {\mathbb {R}}^2$.

The following representation of the normal cone to the set *Z* is obtained in [21, Sect. 4]:

64 $$\begin{aligned} N_Z(\alpha , \beta ) ={\left\{ \begin{array}{ll} \emptyset &{} \quad \text{ if } \quad (\alpha , \beta )\notin Z, \\ \left\{ \alpha \left( \lambda , \mu -\lambda \right) ^\top : \mu \ge 0, \lambda \ge 0 \right\} &{} \quad \text{ if } \quad \alpha \in \left\{ -1, 1 \right\} ,\\ \left\{ \mu \left( \zeta +\alpha , -2 \zeta \right) ^\top : \mu \ge 0 \right\} &{} \quad \text{ if } \quad \alpha \in \left( -1, 1 \right) \wedge \beta \in \left\{ \varphi _1(\alpha ),\varphi _2(\alpha ) \right\} ,\\ \left\{ 0\right\} &{} \quad \text{ if } \quad \alpha \in \left( -1, 1 \right) \wedge \beta \in \left( \varphi _1(\alpha ),\varphi _2(\alpha ) \right) , \end{array}\right. } \end{aligned}$$

where $$\zeta =\text{ sgn }(\alpha -2\beta )$$.

Now, take arbitrarily a vector $\xi = (u,v)^\top \in \mathbb {R}^2$ and observe that $P_Z(\xi )$ is the unique solution of the inclusion

65 $$\begin{aligned} P_Z(\xi ) \in \xi - N_Z(P_Z(\xi )). \end{aligned}$$

Therefore, using the formula (64), one can explicitly calculate $P_Z(\xi )$ as

66 $$\begin{aligned} P_Z(u,v) ={\left\{ \begin{array}{ll} (u,v) &{} \quad \text{ if } \quad (u,v) \in Z,\\ (1,\frac{1}{2}) &{} \quad \text{ if } \quad u \ge 1 \; \text{ and } \; u+v \ge \frac{3}{2}, \\ (-1,-\frac{1}{2}) &{} \quad \text{ if } \quad u \le -1 \; \text{ and } \; u+v \le -\frac{3}{2}, \\ (\alpha _1,\varphi _1(\alpha _1)) &{} \quad \text{ if } \quad u> -1 \; \text{ and } \; u+v< \frac{3}{2}\; \text{ and } \; v< \varphi _1(u), \\ (\alpha _2,\varphi _2(\alpha _2)) &{} \quad \text{ if } \quad u< 1 \; \text{ and } \; u+v < -\frac{3}{2} \; \text{ and } \; v > \varphi _2(u), \end{array}\right. } \end{aligned}$$

where the functions $\varphi _1$ and $\varphi _2$ are defined after (45), $\alpha _1$ is a solution in $[-1,1]$ of the third order equation

67 $$\begin{aligned} \alpha ^3+3\alpha ^2+(9-4v)\alpha -8u-4v-1=0, \end{aligned}$$

and $\alpha _2$ is a solution in $[-1,1]$ of the third order equation

68 $$\begin{aligned} \alpha ^3-3\alpha ^2+(9+4v)\alpha -8u-4v+1=0. \end{aligned}$$

Indeed, the first three cases in the representation (66) are clear. In the fourth case

$$\begin{aligned} u > -1 \; \text{ and } \; u+v< \frac{3}{2} \; \text{ and } \; v < \varphi _1(u), \end{aligned}$$

thus $P_Z(u,v)$ has the form $(\alpha ,\varphi _1(\alpha ))$ (see Fig. 1). From (64), we have

$$\begin{aligned} N_Z((\alpha ,\varphi _1(\alpha )))=\mu (1+\alpha , -2)^\top . \end{aligned}$$

Combining this with (65), one has

$$\begin{aligned} \left( \begin{array}{c} u-\alpha \\ v-\varphi _1(\alpha ) \end{array} \right) =\mu \left( \begin{array}{c} 1+\alpha \\ -2, \end{array}\right) \end{aligned}$$

implying

$$\begin{aligned} \frac{u-\alpha }{v-\varphi _1(\alpha )}= \frac{1+\alpha }{2}, \end{aligned}$$

which leads to (67). The last case is treated similarly.

**3. Solving the auxiliary sub-problem in the CGM**

Now, we consider the subproblem $\min _{y \in K} \, \langle \nabla f^h(w), y\rangle$ which appears in the implementation of the CGM (see (25)).

Observe that, the necessary (and sufficient) optimality condition for this problem reads as

$$\begin{aligned} 0 \in \nabla f^h(w)+N_K (y). \end{aligned}$$

Each component of this inclusion has the form $\left( \xi _1, \xi _2 \right) \in N_Z((\alpha , \beta ))$, which, thanks to (64), can be explicitly represented (see [21]) by the following simple formula:

69 $$\begin{aligned} (\alpha , \beta ) ={\left\{ \begin{array}{ll} \left( -1, -1/2 \right) &{} \quad \text{ if } \quad \xi _1 \le 0 \; \text{ and } \; \xi _1+\xi _2 \le 0, \\ \left( 1, 1/2 \right) &{} \quad \text{ if } \quad \xi _1> 0 \; \text{ and } \; \xi _1+\xi _2 \ge 0, \\ \left( -1-2\xi _1 / \xi _2, \varphi _1(\alpha ) \right) &{} \quad \text{ if } \quad \xi _1> 0 \; \text{ and } \; \xi _1+\xi _2 < 0, \\ \left( 1+2\xi _1 / \xi _2, \varphi _2(\alpha ) \right) &{} \quad \text{ if } \quad \xi _1 \le 0 \; \text{ and } \; \xi _1+\xi _2 > 0. \end{array}\right. } \end{aligned}$$

Therefore, the subproblem (25) can be solved explicitly without solving any third order algebraic equation as in the GPM.

### Numerical Examples

In this subsection, we present some numerical experiments for the example of an affine linear-quadratic optimal control problem given in [24].

#### Example 3.1

70 $$\begin{aligned} \begin{array}{ll} \text{ minimize } &{} -by(1)+ \int _0^1\frac{1}{2}\left( x(t)\right) ^2 {\mathrm{\,d}}t \\ \text{ subject } \text{ to } &{} \dot{x}(t)=y(t), \quad x_1(0)=a \\ &{} \dot{y}(t)=u(t), \quad y(0)=1. \\ &{} u(t) \in [-1,1]. \end{array} \end{aligned}$$

For appropriate values of *a* and *b*, there is a unique optimal solution $\hat{u}$ with a switch from $-1$ to 1 at time $\tau$, which is a solution of the equation

$$\begin{aligned} -5\tau ^4+24\tau ^3-(12a+36)\tau ^2+(24a+20)\tau +24b-12a-3=0. \end{aligned}$$

As in [24], we choose $a=1, b=0.1$, then $\tau =0.492487520$ is a simple zero of the switching function, thus Assumption (A3) is fulfilled. The exact optimal control is

$$\begin{aligned} \hat{u}(t)={\left\{ \begin{array}{ll} -1 &{} \quad \text{ if } \quad t\in [0,\tau ]\\ 1 &{} \quad \text{ if } \quad t\in (\tau , 1]. \end{array}\right. } \end{aligned}$$

For each *N*, the iterates $\left\{ w_k\right\}$ generated by GPM or CGM converge linearly to the unique (in this example) solution $\hat{w}^h$ with rates $\mu _N$ and $\theta _N$, respectively. The starting control is chosen as $u_0(t)=1$, $t \in [0,T]$, for both algorithms. In the following tables, we report these rates for some values of *N*. The stopping condition is $\Vert w_{k+1}-w_k\Vert \le 10^{-6}$ for the GPM and $\Vert x_{k}-w_k\Vert \le 10^{-6}$ for the CGM.

**Table 1:** Convergence rates for the GPM

N	10	20	30	40	50	60	70	80	90	100
$\mu _N$	0.2744	0.4687	0.5742	0.6477	0.6874	0.7166	0.7327	0.8038	0.8736	0.8778

Table 1 indicates that the (numerically obtained) rate of linear convergence, $\mu _N$, of the GPM depends on the mesh size *N*: it is monotone increasing and likely approaching 1 when *N* increases. This is to be expected, since according to Theorem 2.1, the rate $\mu _N$ of linear convergence approaches 1 when $\nu$ goes to zero, and according to Proposition 3.1$\nu$ estimated as proportional to *h*. Actually, the convergence of $\mu _N$ to 1 is also consistent with the fact, that the GPM applied (theoretically) to the continuous-time problem (1)–(3) converges sub-linearly, as recently established in [22, Theorem 3.2]. We emphasize that due to the second order accuracy of discretization, the mesh size *N* does not need to be taken large, therefore the rate of linear convergence may be reasonably good (see Table 1 for $N = 10$–30).

Table 2 presents the rate of linear convergence of the CGM applied to the same example. Although, as mentioned at the end of Sect. 3.4, the amount of computations at each step of the CGM is slightly lower than that for the GPM, the rate of linear convergence is worse.

**Table 2:** Convergence rates for the CGM

N	10	20	30	40	50	60	70	80	90	100
$\theta _N$	0.8946	0.8999	0.9016	0.9023	0.9028	0.9030	0.9032	0.9034	0.9035	0.9036

## Concluding Remarks

In this paper we obtain a number of new results about the convergence of gradient methods for general optimization problems on strongly convex feasible sets. The main motivation is the application of a recently developed discretization scheme [21, 24] for linear-quadratic affine optimal control problems, which results in discrete-time problems of the same type, however, with *strongly convex* point-wise control constraints having rather simple representations by means of quadratic inequalities. This opens several directions of further research.

First, to develop more efficient (than gradient projection) methods using the specific linear-quadratic structure of the objective function and of the constraints.

Second, to investigate the applicability of gradient projection methods to discretized *nonlinear* optimal control problems with the control appearing linearly. As indicated in [17], our discretization approach is also applicable to such problems, and results in mathematical programming problems with strongly convex feasible sets. The general convergence results obtained in the present paper are also applicable, in principle. The main open problem here, is that the error analysis of the discretization is not developed for nonlinear problems, which also creates problems to justify the applicability and the convergence of gradient methods.

## Footnotes

1 The equivalence is proved in [28, Theorem 1] for finite-dimensional Hilbert spaces only, but the proof uses only two-dimensional geometric considerations that work in any Hilbert space.
